# A Systematic Review of Nutrition Interventions for Shift Workers, With Subgroup Analysis of First Responder Populations

**DOI:** 10.1111/jhn.70340

**Published:** 2026-10-01

**Authors:** Alison Jayne Booker, Steven Powell, Joanne Louise Fallowfield, Rachel Gibson

**Affiliations:** ^1^ Ministry of Defence Police, RAF Wyton Cambridgeshire UK; ^2^ Department of Nutritional Sciences King's College London London UK; ^3^ Institute of Naval Medicine Crescent Road Gosport UK; ^4^ Royal Navy People Support Headquarters, HMS Temeraire, Burnaby Road Portsmouth UK

**Keywords:** first responders, nutritional strategies, occupational health, shift work, Systematic review, time‐restricted eating, workplace nutrition

## Abstract

**Introduction:**

Shift work is an increasingly prevalent feature of the modern workforce, with first responders constituting a critical occupational group required to sustain 24‐h operations. Modifiable health behaviour interventions, such as improving diet quality, may be effective in preventing or mitigating the non‐communicable disease onset linked to shift work. This review evaluated evidence supporting nutritional interventions in shift workers with first responder populations considered as a pre‐specified subgroup of interest.

**Methods:**

A systematic review (PROSPERO CRD42023421400) evaluated intervention and observational studies informing dietary guidance for shift workers. Four databases (Embase, Cochrane Library, Web of Science, PubMed) were searched from inception to August 2023, with no date restrictions, and searches were updated in July 2025. Searches were supplemented by grey literature searches specifically targeting first responder populations. Studies were screened by two researchers. Studies of a randomised controlled trial (RCT), crossover‐RCT or observational study design, conducted in free‐living shiftwork populations or a laboratory‐controlled shiftwork setting, the intervention included a dietary component, and health‐related outcomes were reported. A narrative synthesis of results grouped by intervention type was conducted, with a pre‐specified subgroup analysis of studies in first responder populations. Quality assessment was measured using the Cochrane Risk of Bias Tools. Observational studies were evaluated using the Newcastle–Ottawa Scale.

**Results:**

From the 6909 articles retrieved, 43 met the inclusion criteria. Most articles reported using a crossover (49%) or observational (33%) study design. Where specified, most studies involved free‐living participants (67%) in nursing and healthcare professionals (40%), with a few studies (9%) involving first responder subgroups (e.g., firefighters and police officers). Frequently reported intervention components included macronutrient adjustments (23%), time‐restricted eating (TRE) (21%), and meal timing and quantity (19%). Frequently reported outcomes were cardiometabolic health (40%), cognitive performance (30%), and sleep quality (12%). Methodological heterogeneity and prevalent risk of bias limited the evidence strength for determining shift worker nutritional guidelines.

**Conclusion:**

The current evidence‐base is insufficient to formulate evidence‐based dietary guidelines for shift workers. The current evidence base is limited by substantial methodological heterogeneity, small sample sizes, and a moderate‐to‐high risk of bias across studies, which restricts the ability to formulate definitive evidence‐based dietary guidelines for shift workers. While findings may have relevance for high‐risk occupational groups, first responders remain underrepresented in the literature. Future research should prioritise well‐controlled, adequately powered, and ecologically valid intervention studies in diverse shift‐working populations, including first responders, to better inform practical and effective nutritional guidance.

## Practitioner Points

- The evidence for nutritional strategies to support health and performance in shift workers remains limited and heterogeneous.
- Time‐restricted eating and macronutrient distribution strategies show potential benefits for metabolic outcomes, although findings for cognitive and occupational performance are inconsistent.
- First responders are substantially underrepresented, with only four included studies conducted in first responder populations.
- Further high‐quality, pragmatic research is needed to develop nutrition guidance that is evidence‐based and feasible within real‐world shift‐working environments.

## 1 Introduction

Shift work is an increasingly common feature in the workplace, involving work schedules that deviate from the traditional “9 to 5” routine [1] and usually characterised by regular evening or night schedules. The Institute of Employment Rights estimated that 27% of the UK workforce were engaged in night‐time work [2], including critical sectors such as healthcare, manufacturing, and emergency services. Exposure to shift work has been associated with increased incidence of ill‐health [3], metabolic disorders [4] and gastrointestinal conditions [5]. There is also evidence of associations between shift work and impaired lung function [6], reduced muscle mass [7], and fatigue‐related accidents [8]. In first responders, such as police, fire, and ambulance services, these effects pose particular risks, given the high demands for alertness, decision‐making accuracy, and physical capability in unpredictable and hazardous environments [9].

Night shift workers have been shown to adopt less healthful behaviours, such as poorer diet quality. Specifically, studies have indicated shift workers with higher intakes of saturated fat and discretionary foods [10], and lower intakes of fruit, vegetables, and fibre [11, 12]. Evidence also suggests shift work is associated with altered eating patterns, likely due to chronic disruptions in circadian rhythms and structural barriers to maintaining positive health behaviours [13], such as the workplace environment [14]. The misalignment between circadian rhythms and behavioural cycles of eating, sleeping, and physical activity can induce metabolic desynchrony [15], impair glucose tolerance [16], alter appetite‐regulating hormones (leptin and ghrelin) [17], and increase inflammatory signalling [18]. Meal timing and composition may therefore play a central role in mitigating these disruptions, where food intake can act as a zeitgeber (time cue) for peripheral clocks, influencing metabolic pathways in the liver, muscle, and adipose tissue [19].

Modifiable health behaviour interventions, such as improving diet quality, could be effective in preventing, mitigating or delaying the onset of non‐communicable diseases linked to shift work [20]. A study exploring the impact of shift work on nutrition behaviours in UK police officers reported impaired diet quality during shift work, with increased reliance on convenience and poorer‐quality food [21]. This may be attributed to occupational challenges that impact on shift workers' food accessibility, time, and motivation to adopt or maintain health behaviours.

Previous systematic reviews have explored lifestyle and behavioural interventions in shift‐working populations. For example, a recent review examined workplace interventions promoting physical activity and healthy diet behaviours among shift workers [22], while other reviews have evaluated broader non‐pharmacological strategies targeting chronic disease risk factors and sleep outcomes [23]. While these studies provide important insights, they typically consider diet as part of multi‐component interventions rather than as a primary focus, and do not specifically evaluate the effects of nutritional strategies in isolation. Furthermore, there remains limited synthesis of evidence within high‐risk occupational subgroups, such as first responders, who experience unique operational and environmental constraints. Therefore, a focused evaluation of nutritional interventions in shift‐working populations, with specific consideration of first responder groups, is warranted.

Despite growing recognition of these contributing factors, there remains no consensus on evidence‐based dietary guidance for shift workers [24]. This systematic review aimed to evaluate the effects of nutritional interventions on metabolic health, cognitive performance, and health and wellbeing outcomes in shift‐working populations. Specifically, the review sought to (i) identify and characterise the types of nutritional interventions implemented, (ii) examine the range of reported outcomes across metabolic, behavioural, and wellbeing domains, and (iii) assess the extent, nature, and applicability of evidence within first responder populations as a pre‐specified subgroup. The review also aimed to identify key methodological limitations and research gaps to inform future study design and dietary guidance.

## 2 Methods

### 2.1 Search Strategy

The systematic review protocol was registered with the International Prospective Register of Systematic Reviews (PROSPERO) registration number CRD42023421400 and was reported according to the Preferred Reporting Items for Systematic Reviews and Meta‐Analyses (PRISMA) framework [25]. The Population, Intervention, Comparison, Outcomes, and Study design (PICOS) [26] approach was used to define the research question, develop the search terms, and determine study eligibility. The search strategy was developed by AB, RG, SP and JF with support from King's College London and the College of Policing National Library Librarians.

Search terms were developed using a combination of Medical Subject Headings (MeSH) and free‐text keywords relating to shift work (e.g., “shift work”, “night work”, “rotating shifts”) and dietary factors (e.g., “diet”, “nutrition”, “eating patterns”). Boolean operators, truncation, and wildcards were applied as appropriate to capture variations in terminology. An example search strategy for Cochrane, SCOPUS, and Web of Science is provided in Supporting Information S1: Table S1, with full search strategies included to enhance transparency and reproducibility. Grey literature search strategies were developed using similar keyword combinations and applied across the ProQuest database, clinical trial registries, and institutional resources (College of Policing).

### 2.2 Eligibility Criteria

Studies were included if they involved human adults, including free‐living shift workers working night shifts or participants undertaking simulated shift work in laboratory settings (Population). Eligible studies included a dietary component, defined as diet quality or eating patterns (Intervention/Exposure), and were compared with usual diet, habitual intake, or existing dietary recommendations where applicable (Comparator). Studies were required to report at least one health‐related outcome, including physiological (e.g., metabolic or gastrointestinal health), behavioural (e.g., cognitive or occupational performance), or wellbeing‐related outcomes (e.g., sleep, quality of life, mood, hunger) (Outcomes). Eligible study designs included randomised controlled trials, crossover trials, and observational studies reporting a dietary exposure and outcome(s) (Study design). Subgroup analysis of first responder populations (e.g., police, fire, and ambulance personnel) was conducted where data were available.

Studies were excluded if they involved non‐human (animal or in vitro) populations, paediatric populations, or did not include a dietary component or relevant health‐related outcome. Studies focusing exclusively on fluid or caffeine interventions were excluded *a priori* to avoid duplication with an ongoing registered systematic review in this area (PROSPERO CRD42024509202). In addition, studies not published in English, conference abstracts without full‐text availability, review articles, and other non‐primary research were excluded.

### 2.3 Literature Searches

A comprehensive literature search in Cochrane Library (CENTRAL), Web of Science, PubMed, and SCOPUS was conducted from database inception to August 2023, with no date restrictions applied, using Medical Subject Headings (MeSH) terms and relevant keywords to identify studies that reported dietary interventions in shift working populations. The search terms were adapted for each database to account for differences in indexing and search functionalities. Truncation and wildcards were used to broaden the search and capture variations of the search terms. The initial search was restricted to title, abstract and keywords. Consideration was given to first‐responder occupations, but a relatively small number of studies (*n* 16) met the initial eligibility criteria in these occupational groups. Grey literature sources were systematically searched, including clinical trial registers (ClinicalTrials.gov), a physical search at the National Police Library, College of Policing MyAthens, and the ProQuest database. Informal interviews were conducted with senior representatives from the UK Ambulance Service and Fire Service during May 2023 via Microsoft Teams to identify any additional repositories of grey literature. Snowball searching was performed from the reference lists of all included papers, ensuring searches for studies was as extensive as possible to reduce the risk of publication bias and to identify as much relevant evidence as possible. Searches were re‐run on 14 July 2025 to identify any additional relevant studies published since the initial search.

Included papers were managed and screened using the online Systematic and Literature Review platform Rayyan (https://www.rayyan.ai). After the removal of duplicate publications, two reviewers (AB, RG) independently screened all titles and abstracts to determine the eligibility of each study. A third reviewer (SP) resolved disagreements through discussion and consensus. Full texts were then screened against the predefined inclusion and exclusion criteria by two researchers (AB, SP) with a third reviewer (RG) resolving disagreements.

### 2.4 Data Extraction and Synthesis

A standardised data extraction table was developed a priori in Microsoft Excel to ensure consistency and completeness. Data were extracted from each of the included studies, including details of study design, type of shift work, occupational groups, participant characteristics, health status plus co‐morbidities, type and duration of intervention, and primary and secondary outcomes. Where studies did not include a discrete intervention, relevant dietary or experimental exposure (e.g., dietary inflammatory index or simulated shift timing) data were extracted and analysed alongside intervention studies. Data extraction was conducted independently by AB and SP.

### 2.5 Analysis

Due to heterogeneity of interventions, outcome measures, and reporting practices, a meta‐analysis was not possible [27]. Thus, the systematic review without meta‐analysis (SwiM) reporting guidelines were followed [28]. Findings were narratively synthesised by intervention type (macronutrient composition, TRE, diet quality), and primary outcomes (metabolic, health, performance, sleep quality), to show overall trends in the literature. Where appropriate, outcomes were categorised as metabolic or cardiometabolic based on the nature of the physiological markers assessed. A planned subgroup analysis was conducted for studies involving first responder populations to explore the applicability of findings to these occupational groups, given their high prevalence of shift work and unique operational demands.

### 2.6 Risk of Bias Assessments

Risk of bias was determined across multiple domains using the Cochrane Risk of Bias Tools for RCT [29] and C‐RCT [30] study designs. Observational studies were evaluated using the Newcastle–Ottawa Scale [31]. Each study was assessed for critical, high, moderate, or low risk of bias within each of the specified risk domains and an overall grade assessment was allocated. A sub‐group analysis was conducted in the papers involving first responder populations due to the longer‐term aims of the authors following this systematic review and the high prevalence of shift working in these occupations. The Risk of Bias assessment was conducted by AB.

## 3 Results

### 3.1 Search Results

From the initial database search, 10,054 studies were identified with an additional 631 studies identified from grey literature searching. In total, 3759 duplicates were removed and 6909 abstracts from the initial searches were screened, in addition to 17 abstracts from the hand search and citation searching. Of these, 11 papers were not accessible in full text form, and 87 papers were excluded (Figure 1). After full text screening, 43 papers met the inclusion criteria. Most included studies were conducted in general shift‐working populations, particularly healthcare workers, with a smaller subset (*n* 4) involving first responder groups.

**Figure 1:**
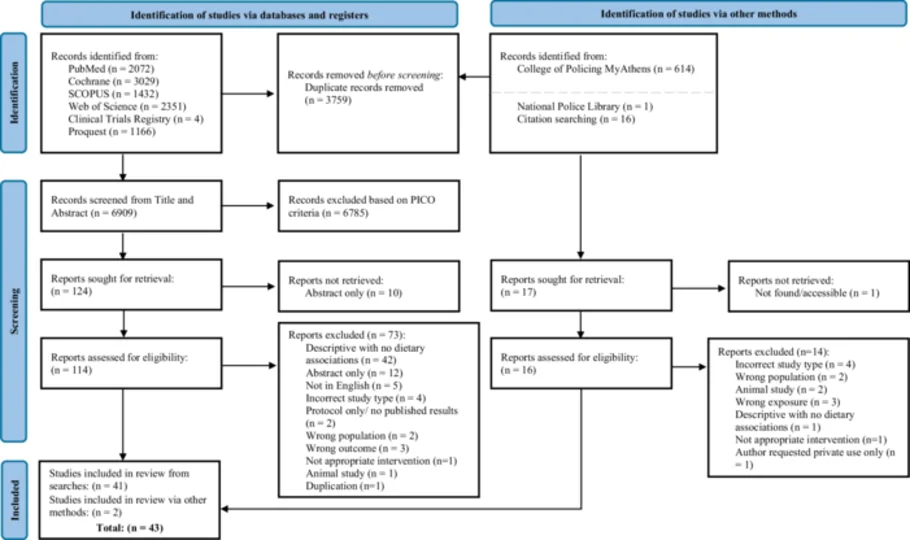
PRISMA 2020 flow diagram for new systematic reviews which included searches of databases, registers, and other sources.

The re‐run of search terms on 14 July 2025 found 6 additional papers published between August 2023 and July 2025 that met the inclusion criteria. Analysis of these papers corroborated the overarching themes drawn from the original analysis, with no significant developments or changes to the overall conclusions.

### 3.2 Study and Population Characteristics

Of the 43 studies included, 21 (49%) were C‐RCT, 14 (33%) were observational studies, and 8 (19%) were RCT. Of the included studies, 10 (23%) were conducted in North America, 10 (23%) in Europe and Scandinavia, 9 (21%) in Australia and New Zealand, 5 (12%) in South America, 4 (9%) in Asia, 3 (7%) in the Middle East, and 2 (5%) in the UK. Ethnicity of participants is not widely reported, with 34 (79%) studies not providing specific data. The mean percentage of individuals of white ethnicity varied across study types, specifically 79% in RCT, 27% in C‐RCT, and 22% in observational study designs. A high proportion of studies involved free‐living participants (*n* 28, 65%) and were carried out with nursing and healthcare professionals (*n* 17, 40%). Only 4 (9%) studies were conducted in first responder groups.

Across studies, over 20 different definitions of night shift timing were identified. For clarity, these were grouped into broader categories based on whether timing was specified, not defined, or experimentally manipulated (Table 1). Shift type was reported in 34 (79%) of the 43 included studies. Of these, 11 (26%) involved rotating shift workers and 5 (12%) involved fixed night shift workers. A substantial proportion of studies were conducted in simulated shift work settings (*n* 15, 35%), while 3 (7%) studies included mixed shift patterns. Nine (21%) studies did not specify shift type. The variability and incomplete reporting of shift patterns may limit interpretation of the influence of shift type on dietary behaviours, particularly in occupational groups such as first responders where rotating schedules are common. Years in shift work were often not reported (*n* 17, 40%) and, likely due to the prevalence of simulated protocols, a moderate number of studies involved participants with no shift work experience (*n* 12, 28%). As such, 14 (33%) studies reported shift work experience among participants, of which 10 (23%) studies involved participants with 5 years or less, 1 (2%) study involved participants with between 6 and 10 years, and 3 (7%) studies reported an average of 11+ years of shift work experience.

**Table 1:** Characteristics of included studies by study design.

	All (*n* 43)	RCT (*n* 8, 19%)	C‐RCT (*n* 21, 49%)	Observational (*n* 14, 33%)
Country				
US and Canada	10 (23%)	5 (63%)	1 (5%)	4 (29%)
Europe incl Scandinavia	10 (23%)	0 (0%)	6 (29%)	4 (29%)
Australia and NZ	9 (21%)	1 (13%)	8 (38%)	0 (0%)
South America	5 (12%)	1 (13%)	3 (14%)	1 (7%)
Asia	4 (9%)	0 (0%)	1 (5%)	3 (21%)
Middle East	3 (7%)	1 (13%)	0 (0%)	2 (14%)
UK	2 (5%)	0 (0%)	2 (10%)	0 (0%)
Occupation				
Nursing and Healthcare	17 (40%)	4 (50%)	5 (24%)	8 (57%)
Simulation participants	13 (30%)	1 (13%)	11 (53%)	1 (7%)
First Responders	4 (9%)	1 (13%)	1 (5%)	2 (14%)
Factory/Industrial worker	4 (9%)	0 (0%)	2 (10%)	2 (14%)
Student	2 (5%)	0 (0%)	2 (10%)	0 (0%)
Security	2 (5%)	1 (13%)	0 (0%)	1 (7%)
Mixed	1 (2%)	1 (13%)	0 (0%)	0 (0%)
Shift work definition (timing/duration)				
Night shift (timing specified)	26 (61%)	4 (50%)	16 (76%)	6 (43%)
Night shift (timing not defined)	3 (7%)	4 (50%)	2 (10%)	1 (7%)
Not defined	14 (33%)	0 (0%)	3 (14%)	7 (50%)
Shift pattern				
Simulated	15 (35%)	3 (38%)	11 (52%)	1 (7%)
Rotating	11 (26%)	3 (38%)	2 (10%)	6 (43%)
Not specified	9 (21%)	1 (13%)	3 (14%)	5 (36%)
Fixed	5 (12%)	1 (13%)	2 (10%)	2 (14%)
Mixed	3 (7%)	0 (0%)	3 (14%)	0 (0%)
Years in (night) shift work				
Not reported	17 (40%)	2 (25%)	6 (29%)	9 (64%)
Zero	12 (28%)	1 (13%)	11 (52%)	0 (0%)
Average 0–1 years	7 (16%)	3 (38%)	2 (10%)	2 (14%)
Average 2–5 years	3 (7%)	1 (13%)	1 (5%)	1 (7%)
Average 11+ years	3 (7%)	1 (13%)	0 (0%)	2 (14%)
Average 6–10 years	1 (2%)	0 (0%)	1 (5%)	0 (0%)
Participant characteristics (mean)				
No. participating (range)	119 (6–1638)	45 (9–137)	18 (6–61)	312 (7–1638)
Age, years (SD)	33.2 (5.6)	33.0 (6.2)	31.3 (5.2)	36.1 (5.9)
Sex, % male (range)	43% (0–603)	67% (0–125)	75% (0–40)	39% (0–603)
Ethnicity, % white	26%	79%	27%	22%
BMI, kg/m2 (SD)	25.9 (3.6)	26.7 (4.0)	25 (3.2)	26.9 (4.2)
Health status, % Healthy	79%	75%	86%	71%

*Note:* Percentages rounded to whole numbers.

The mean number of participants across all studies was 119, of which for RCT the mean was 45 (range 9 to 137), for C‐RCT 18 (range 6 to 61) and observational studies 312 (range 7 to 1638). The mean participant age was 33 years (SD 5.6) and was similar across the RCT and C‐RCT study types. The mean age of participants in observational studies was 36 years (SD 5.9). Studies were often conducted in all‐male groups (*n* 14, 33%) or all‐female groups (*n* 9, 21%). Overall, the percentage of males in all studies was 43%, this differed by study type with C‐RCTs representing the highest percentage of male participants (74%), followed by RCTs (67%) and then observational study designs (39%). The mean body mass index (BMI) of participants across all studies was 25.9 kg/m^2^ (SD 3.6). A breakdown of study participant characteristics are presented in Table 1. A full list of study characteristics are presented in Supporting Information S1: Table S2.

### 3.3 Characteristics of Nutrition Interventions

Dietary interventions most frequently involved modification of macronutrient composition (*n* 10, 23%) [32, 33, 34, 35, 36, 37, 38, 39, 40, 41], including manipulation of carbohydrate, protein, and fat intake (e.g. high‐carbohydrate, high‐protein, or altered fat‐content diets), alongside time‐restricted eating (TRE) (*n* 9, 21%) [42, 43, 44, 45, 46, 47, 48, 49, 50], and meal timing interventions (*n* 8, 19%) [51, 52, 53, 54, 55, 56, 57]. One study (2%) adopted nutritional counselling as an intervention [58] to support improvements to food choices and eating patterns for participating shift workers. Where reported, dietary interventions were delivered by a range of providers, including dietitians and research staff. However, reporting of intervention delivery was inconsistent across studies, limiting the ability to draw firm conclusions regarding the influence of delivery approach on outcomes. Intervention intensity also varied, including differences in duration, frequency, and level of participant support.

Overall diet quality and dietary patterns were considered in 10 studies (23%), which utilised a variety of interventions/exposures. These included the consumption of specific food groups (*n* 3, 7%) [59, 60, 61] and sugar intake (*n* 2, 5%) [62, 63], broader dietary patterns (*n* 2, 5%) [64, 65], meal frequency (*n* 1, 2%) [66], eating speed and energy (kcal) distribution (*n* 1, 2%) [59], improving healthy food choices in the workplace (*n* 1, 2%) [67], and pro/pre‐biotic intake (*n* 1, 2%) [68]. In addition, three studies consider the dietary inflammatory index (DII) as either an exposure (*n* 2, 5%) [69, 70] or outcome (*n* 1, 2%) [55] plus one study measured inflammatory biomarkers [64].

### 3.4 Reported Outcomes

Twenty studies (29%) reported outcomes related to metabolic health such as glucose metabolism, insulin sensitivity, and lipid profiles. A further 15 studies (22%) considered cardiometabolic health, incorporating measures of blood pressure, body composition, and cardiovascular risk markers. The same number of studies (*n* 15, 22%) examined cognitive and performance outcomes, assessing domains such as attention, reaction time, and occupational performance in relation to nutritional interventions or eating schedules.

A smaller subset evaluated sleep‐related outcomes (*n* 5, 7%) including sleep duration, quality, and circadian alignment. Diet quality was quantified in 4 studies (6%), typically assessing adherence to healthy eating guidelines or improvements in nutrient intake. Fewer studies reported subjective outcomes, such as gastrointestinal (GI) complaints (*n* 3, 4%), quality of life (QoL) (*n* 3, 4%), hunger or appetite regulation (*n* 3, 4%) and mood (*n* 1, 2%). A breakdown of intervention characteristics and reported outcomes are presented in Table 2. Full details of included interventions in Supplementary Table S3.

**Table 2:** Characteristics of interventions and reported outcomes.

	All (*n* 43)	RCT (*n* 8, 19%)	C‐RCT (*n* 21, 49%)	Observational (*n* 14, 33%)
Intervention/Exposure				
Diet quality/Dietary patterns	10 (23%)	1 (2%)	2 (5%)	7 (16%)
Macronutrient adjustment	10 (23%)	3 (7%)	6 (14%)	1 (2%)
Time Restricted Eating	9 (21%)	3 (7%)	6 (14%)	0 (0%)
Meal timing	9 (21%)	0 (0%)	6 (14%)	3 (7%)
Inflammatory index	2 (5%)	0 (0%)	0 (0%)	2 (5%)
Simulated shift timing	2 (5%)	1 (2%)	0 (0%)	1 (2%)
Nutritional counselling	1 (2%)	0 (0%)	1 (2%)	0 (0%)
Reported outcomes				
Metabolic Health	20 (29%)	5 (7%)	8 (19%)	7 (16%)
Cardiometabolic health	15 (22%)	3 (4%)	6 (14%)	6 (14%)
Cognition and Performance	15 (22%)	2 (5%)	8 (19%)	5 (7%)
Sleep quality	5 (7%)	1 (2%)	0 (0%)	4 (6%)
Diet Quality	4 (6%)	0 (0%)	3 (4%)	1 (2%)
GI Complaints	3 (4%)	0 (0%)	3 (4%)	0 (0%)
QoL	3 (4%)	1 (2%)	2 (5%)	0 (0%)
Hunger	3 (4%)	0 (0%)	3 (4%)	0 (0%)
Inflammatory markers	1 (2%)	0 (0%)	0 (0%)	1 (2%)
Mood	1 (2%)	0 (0%)	0 (0%)	1 (2%)

*Note:* Percentages rounded to whole numbers. Metabolic health outcomes included measures of glucose regulation and lipid metabolism (e.g., glucose, insulin, triglycerides, cholesterol). Cardiometabolic health outcomes additionally included cardiovascular‐related markers such as blood pressure and composite risk indices.

### 3.5 Sub‐Group Analyse

#### 3.5.1 Macronutrient Interventions

Interventions modifying macronutrient composition varied considerably across studies. Where reported, higher protein diets ranged from approximately 30%–60% of total energy intake, while higher fat interventions typically provided ~40%–45% of total energy. Carbohydrate content varied widely depending on study design, ranging from ~20% to 70% of total energy intake across comparison conditions. As such, classifications such as “high” or “low” macronutrient content are reported relative to the study‐specific conditions rather than fixed thresholds. Carbohydrate content was the most altered macronutrient (*n* 9, 21%), of which 5 (12%) studies implemented high‐carbohydrate diets (approximately 60%–70% of total energy), while 3 (7%) examined increased carbohydrate intake relative to baseline or a control condition. One study (2%) considered both low and high carbohydrate conditions, also adjusting for low and high protein intake. A high protein condition (approximately 30%–60% of total energy intake across studies) was the focus of 3 (7%) studies, and 3 (7%) studies focused on a higher dietary‐fat condition (typically ~40%–45% of total energy intake). An overview of the interventions that trialled macronutrient compositions is at Table 3.

**Table 3:** Macronutrient composition and outcomes.

Study	Study type	Simu‐lated shift work	Macronutrient Composition			Primary outcomes	Method	Description	Intervention effects on primary outcome	Mean difference	*p* value
			Iso‐caloric	Control/Cond 1 (% Kcal)	Condition 2 (% Kcal)						
Bonham et al., 2020 [4]	C‐RCT	Y	Y	13% CHO 25% fat	28% CHO 45% fat	Post‐prandial glucose	Serum	iAUC	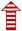	—	0.04
						Post‐prandial insulin			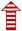	—	0.02
						Post‐prandial triglyceride			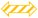	—	0.72
Cunha et al., 2020 [7]	RCT	N	Y	20% Pro 60% CHO 20% fat	60% Pro 20% CHO 20% fat	Post‐prandial glucose	Serum	iAUC	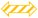	—	0.712
						Post‐prandial insulin			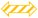	—	0.732
						Post‐prandial triglyceride			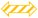	—	0.426
HolmbÃck et al., 2003 [16]	RCT	Y	Y	40% CHO 45% Fat	65% CHO 20% Fat	Glucose	Serum	ANOVA	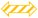	—	—
						Insulin			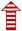	—	0.028
						Cortisol			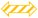	—	—
						Growth hormone			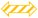	—	—
						Lipid metabolism			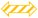	—	—
Love et al., 2005 [25]	C‐RCT	N	N	56% CHO 28% fat	46% CHO 42% fat	Sleep	Subjective Digital thermometer Reaction time	Sleep scale ^o^C PASAT Score*	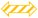	0.5 ± 0.8	—
						Body temperature			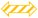	−0.37	—
						Cognitive			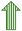	5.0 ± 1.5	0.042
Lowden A et al., 2004 [26]	C‐RCT	Y	Y	15% Pro 40% CHO 45% Fat	15% Pro 65% CHO 20% Fat	Sleepiness	Karolinksa Drowsiness Test	EEG**	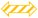	—	—
						Cognitive	Auditory reaction time	ANOVA	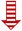	—	0.01
Lowden et al., 2001 [27]	Obs	Y	Y	15% Pro 40% CHO 45% Fat	15% Pro 65% CHO 20% Fat	Mood	Subjective scale	ANOVA	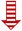	1.28 ± 0.04	—
						Hunger			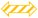	—	—
Makowski et al., 2022 [30]	RCT	N	NR	25% Pro 40% CHO 35% Fat	12% Pro 54% CHO 34% Fat	Psychomotor Vigilance	Neuro‐behavioural Tests	Scale	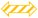	—	0.087
						Sensory‐motor Speed			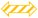	—	0.583
						Working Memory			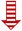	—	< 0.001
						Risk Decision Making			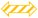	—	0.081
Nehme et al., 2014 [33]	RCT	N	N	CHO Condition: plus 20%–30% on baseline	Pro Condition: plus 30%–40% on baseline	Sleep duration	Actigraphy	ANOVA	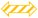	—	0.98
Paz and Berry., 1997 [37]	RCT	N	N	CHO Condition: 4% Pro 70% CHO 26% fat	Pro Condition: 52% Pro 20% CHO 28% fat	Alertness	Stanford scale	Subjective mg/dL μU/mL	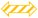	—	0.07
						Cognitive	Alertness		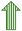 ***	—	0.05
						Post‐prandial glucose	Serum		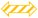	—	—
						Post‐prandial insulin			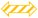	—	—
Silva et al., 2020 [39]	C‐RCT	N	Y	15% Pro 65% CHO 20% Fat"	35% Pro 45% CHO 20% Fat"	Appetite	Subjective scale	ANOVA	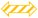	—	0.252
						Satiety			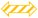	—	0.283

*Note:* See colour code key, below. Arrows indicate direction of significance of intervention on primary outcome. *EEG: electroencephalogram. **PASAT: Paced Auditory Serial Addition Test. ***Statistically significant in high protein intervention diet.

Overall, evidence across studies consistently indicated that higher carbohydrate intake during night shifts resulted in adverse post‐prandial metabolic responses, whereas higher protein interventions showed some evidence of improved alertness, although metabolic effects were inconsistent. Results also indicated significantly higher post‐prandial insulin concentrations compared with daytime feeding or lower‐carbohydrate diets [32, 64]. A high protein intervention diet resulted in a significant improvement in alertness scores [39], although results for metabolic markers did not reach statistical significance [33, 39].

Evidence for high‐fat interventions was limited and inconsistent. Although one study reported improvements in cognition and reaction time following higher‐fat meals [35], others showed negative effects to reactions and mood [36, 41]. Cognitive and psychomotor outcomes varied considerably between studies, potentially reflecting differences in meal composition, timing, or task demands.

### 3.6 TRE Schedules

Nine studies (21%) using a TRE intervention were evaluated, all of which employed either a RCT or C‐RCT design. Primarily these studies were conducted within controlled laboratory settings designed to simulate night‐shift conditions. Most adopted a “no eating at night” (NEN) approach (*n* 8, 19%), restricting food intake between approximately 01:00 and 06:00 h, while one study permitted participants to self‐select a 10‐h fasting window (mean start 19:00, end 09:00). Intervention durations ranged from single‐night laboratory trials to multi‐week free‐living protocols. An overview of TRE interventions is at Table 4.

**Table 4:** Time restricted eating (TRE) schedules and outcomes.

Study	Study type	TRE Schedule	Simulated shiftwork	Primary outcomes	Method	Description	Effect on primary outcome(s)	Mean difference	*p* value
Chellappa et al., 2021 [6]	RCT	NEN v No restriction	Y	Circadian misalignment	Melatonin (saliva)	Phase shift	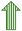	1hr 51min	< 0.004
				Glucose tolerance	Serum	iAUC	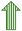	77.1 ± 24.6 mg/dL	0.003
Grant et al., 2017a [9]	RCT	NEN v Snack at 01:30	Y	Cognitive performance	Cognitive tests	Cognition	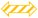	—	—
				GI discomfort	Subjective	Gut reaction scale	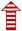	—	0.004
Grant et al., 2017b [10]	RCT	NEN v Snack at 01:30	Y	Post‐prandial glucose	Serum	iAUC*	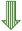	—	0.001
				Post‐prandial insulin		iAUC	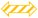	—	0.825
Gupta et al., 2017 [11]	C‐RCT	NEN v Meal at 01:30	Y	Driving performance	Simulation tests	Safe zone	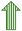	—	< 0.001
						Speed variability	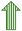	—	
						Lane variability	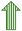	—	
Gupta et al., 2021 [12]	C‐RCT	NEN v Meal or snack at 00:30	Y	Driving performance	Simulation tests	Safe zone	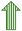	—	0.04
						Speed variability	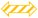	—	—
						Lane variability	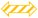	—	—
Leung et al., 2021 [24]	C‐RCT	No eating between 01 ‐ 0600	N	Post‐prandial Triacylglycerol	Serum	iAUC	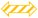	−0.5 AUC (95% CI ‐1.9, 0.9)	0.47
				Post‐prandial glucose			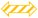	0.3 AUC (95% CI ‐0.7, 1.2)	0.63
				VLDL Cholesterol Particle size			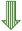	−1.34 (−2.20–−0.49)	0.003
				HbA1c**			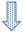	−0.31 (−0.70–0.08)	0.012***
Manoogian et al., 2022 [29]	RCT	Reduced eating window to 10hrs	N	TRE Feasibility	Questionnaires/interviews	Eating window	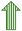	95% CI 10.7–11.5 h	3.29E‐17
				Fasting glucose	Serum	Fasting levels	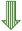	95% CI −0.41 to −0.01 mmol/L	0.043
				VLDL Cholesterol Particle size			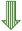	−1.34 (−2.20–−0.49)	0.003
Oriyama and Yamashita., 2021 [35]	C‐RCT	NEN v Meal at 03:30	Y	Cognitive performance	Psychomotor vigilance task	Cognition	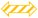	—	—
				Sleepiness	Subjective scale	Alertness	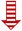	—	0.659
Teixeira et al., 2022 [41]	C‐RCT	NEN v Meal at 02:00	N	Next day eating	Food diaries	Sugar intake	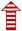	19.1g (95% CI, −36.2 to −2.1)	< 0.05
				Post‐prandial glucose	Serum	iAUC	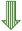	—	< 0.05

*Note:* See colour code key, below. Arrows indicate direction of significance of intervention on primary outcome. *iAUC: incremental Area Under the Curve. **HbA1c: glycosylated haemoglobin. *** P value is only significant where baseline levels indicate elevated start levels.

**Key**
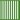
   Statistically significant, TRE beneficial
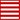
   Statistically significant, TRE non‐beneficial
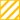
   Not statistically significant
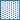
   Statically significant, only where baseline levels indicate poor health

### 3.7 First Responder Populations

First responder populations were the focus of four (9%) of the included studies; one (2%) was conducted with firefighters and utilised a RCT study design, the remaining three (7%) were conducted with police officers and were made up of two observational studies and a C‐RCT. Of these four studies, three were conducted in North America (1 in firefighters, 2 in police officers) and 1 was conducted in South America (police officers).

Only two papers reported shift work experience; one reported an average of 16 years (SD 8.3) among 447 (75% male) police officers [70] while another reported an average of 3 years (SD 1.4) among 10 (100% male) police officers [50]. Firefighters were the subject of just 1 study, of which 137 (91% male) firefighters participated [48] (Supplementary Table S8).

### 3.8 Strength of Evidence – Risk of Bias

The risk of bias assessment highlighted 68% of the included papers were judged to be of concern or moderate risk of bias (*n* 8, 19%), a serious or high risk of bias (*n* 17, 40%), or critical risk of bias (*n* 4, 9%). Figure 2 provides an overview of the overall risk of bias classification by study design. Supporting Information S1: Tables S9–S11 provide a detailed overview, per domain.

**Figure 2:**
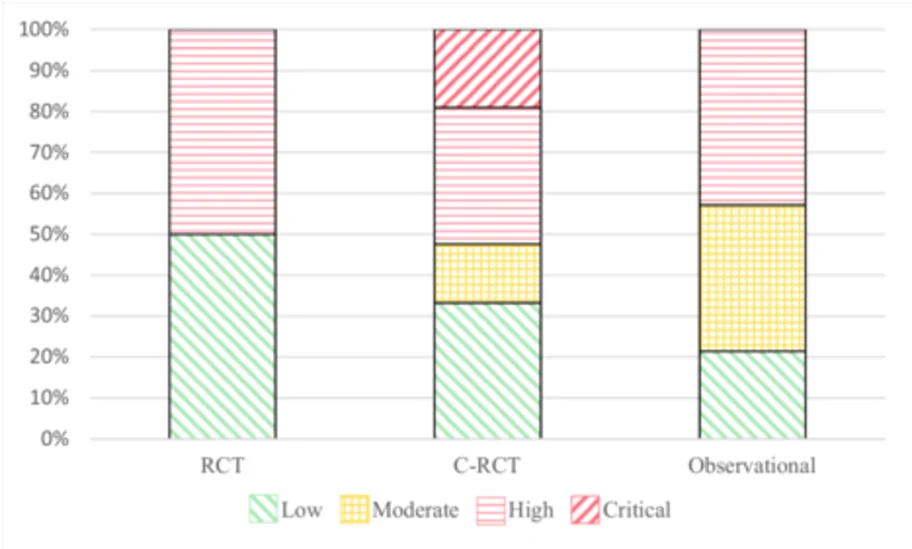
Risk of bias assessment by study design.

For C‐RCTs (*n* 21) assessed using the ROBINS‐I V2 tool (S8), most studies displayed *some concerns to high risk of bias* (*n 15*, 71% C‐RCT*)*, particularly in the confounding domain (*n* 10). Generally, these studies also demonstrated concerns in outcome measurement, which impacted their overall risk of bias score. Missing data bias and result selection bias were additional sources of uncertainty. Intervention classification and participant selection were generally well managed across studies with a subset (*n* 6) demonstrating a *low risk of bias* across all domains (Figure 3).

**Figure 3:**
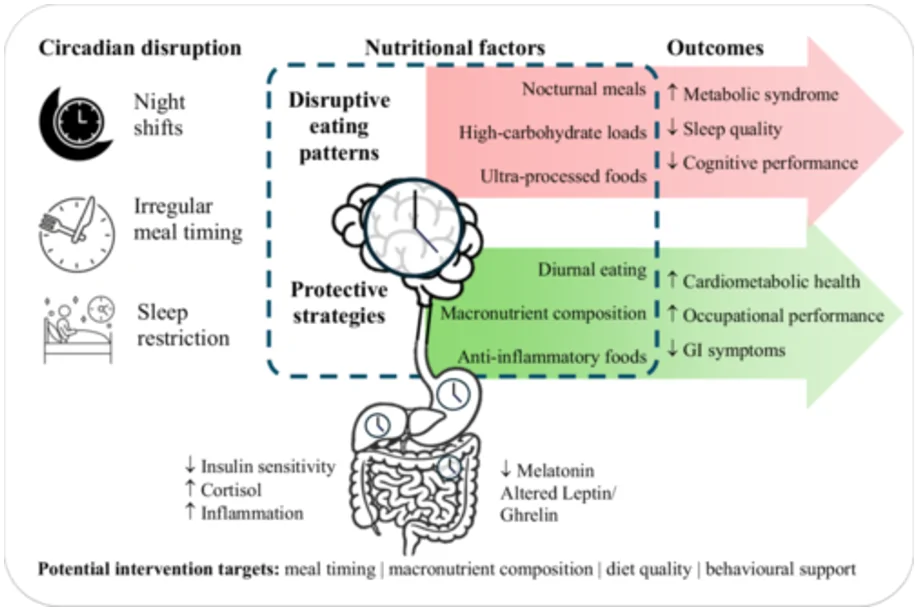
Conceptual model illustrating nutrition as a modulator of circadian and health outcomes in shift work. Schematic illustrates the proposed mechanisms linking nutritional behaviours, circadian disruption, and health outcomes in shift working populations. It presents two main pathways, disruptive versus protective, showing how eating patterns influence circadian alignment, metabolic function, and performance.

Across observational studies (*n* 14) evaluated using the Newcastle–Ottawa Scale (S9), methodological quality ranged from *poor to fair* (*n* 11), with few studies achieving a *good* rating (*n* 3). The selection and outcome domains were most frequently under‐rated, often due to limited control for confounders or inadequate description of participant recruitment. Studies scoring more highly tended to provide clearer outcome measures and longitudinal data, though heterogeneity in exposure assessment remained a limitation.

In contrast, RCTs (*n* 8) assessed via the Cochrane Risk of Bias tool (S11) demonstrated the lowest overall risk of bias. Most RCTs were rated as *low risk or having only minor concerns*, particularly regarding randomisation and deviations from intended interventions. However, the outcome measurement and selective reporting domains presented occasional concerns, often due to small sample sizes or incomplete reporting of outcomes.

## 4 Discussion

Shift work is consistently associated with a higher incidence of non‐communicable conditions [3, 5]. Notably, shift workers often adopt less healthful dietary behaviours, including higher intakes of saturated fat, coupled with lower consumption of fruits, vegetables, and fibre [10, 11, 12]. The role of nutrition and diet is therefore likely to be important in mitigating some of this risk, as meal timing and composition can play a role in counteracting metabolic disruptions and influencing peripheral clocks [19]. Despite the growing recognition of these mechanisms, there remains no consensus on evidence‐based dietary guidelines for shift workers.

This systematic review aimed to evaluate and synthesise the available evidence from both observational and interventional studies examining nutritional strategies in shift‐working populations, with a focus on the range of interventions implemented and the associated health, wellbeing, and performance outcomes. Particular attention was given to first responder groups due to the hypothesised risk posed by shift work in these occupations, given the critical demands for alertness, accurate decision‐making, and physical capability in unpredictable and sometimes hazardous environments. The interplay between nutrition, circadian disruption, and operational readiness is critical for this population, yet first responders remain underrepresented in the literature.

This review found that, across studies, TRE and macronutrient distribution strategies emerged as promising approaches to improving metabolic outcomes. In particular, the findings indicate that TRE; particularly strategies that eliminate food intake during the biological night, may support metabolic and circadian alignment [42]. TRE studies were shown to have beneficial effects on glucose tolerance [42, 43, 48, 50] and serum cholesterol [47, 48] when compared to unrestricted and/or nocturnal eating [55]. One study demonstrated that NEN reduced circadian misalignment by nearly 2 h and improved post‐prandial glucose excursions [42], while others observed favourable effects on very‐low‐density lipoprotein (VLDL) cholesterol particle size [47, 48] and glycosylated haemoglobin (HbA1c) among participants with elevated baseline levels [47]. The research shows that the timing of food intake has a critical effect on metabolic parameters during shift work, indicating that TRE‐based strategies may represent a practical and targeted approach to supporting metabolic health in these populations [71, 72].

Across the trials, higher carbohydrate intake during night shifts was consistently associated with elevated post‐prandial glucose and insulin responses. Results also indicated significantly higher post‐prandial insulin concentrations compared with daytime feeding or lower‐carbohydrate diets [32, 64]. A high protein intervention diet resulted in a significant improvement in alertness scores [39], although results for metabolic markers did not reach statistical significance [33, 39].

These findings suggest that meal‐timing and dietary composition interact with circadian physiology, offering opportunities to tailor nutritional strategies to minimise metabolic strain during shift work. However, the operational realities of first responder roles could present some barriers to implementing TRE and strict macronutrient protocols in practice. Unpredictable callouts, limited control over meal breaks, and rotating night shifts could make adherence to fixed eating windows and meal preparation difficult [53, 73]. Environmental and logistical constraints, including prolonged operational demands, may also challenge the feasibility of maintaining nutrient‐dense, time‐limited eating patterns. Nevertheless, one study found that TRE was feasible and well‐tolerated among firefighting shift‐working cohorts, suggesting that with appropriate flexibility, such approaches may be adaptable to operational contexts [48].

However, the evidence base remains inconsistent for cognitive and occupational performance outcomes. While some studies report improvements in reaction time and vigilance under TRE conditions [45, 50], others observed no significant effects or even decrements in alertness [43, 46, 49]. One study reported significant decrements in reaction times and memory task in the NEN condition, however there were significant improvements in cognition where participants consumed a snack at night (10% kcal) [53]. Potentially, snack‐based nightshift eating could represent a promising but under‐explored approach.

Further, while one study indicated metabolic advantages to TRE, it also found an association between TRE and poorer nutritional behaviours during subsequent eating windows [50]. This finding contradicts established diet quality research for diet and long‐term health outcomes [74]. In addition, one TRE study reported negative effects on gastrointestinal discomfort [43]. This is pertinent given that epidemiological studies consistently demonstrate a heightened incidence of GI related complaints among shift workers [75]. Consequently, the short‐term nature of many TRE studies, the small pool of studies that capture GI symptoms during the night plus dietary habits on days following fasting windows, is a current limitation. Strategies such as TRE must strike a careful balance between enhancing metabolic health, sustaining night‐shift performance, and maintaining worker comfort and acceptability.

Alternative macronutrient distribution strategies showed some promise for modulating metabolic responses and cognitive performance in shift workers. Higher protein, moderate‐carbohydrate diets (approximately 30%–60% protein and ~40%–50% carbohydrate) tended to support favourable outcomes, while high‐carbohydrate meals (approximately 60%–70% carbohydrate) consumed during the night elevated glycaemic and insulin responses [32, 33, 34, 40]. However, high‐protein diets did not always reach statistical significance for metabolic markers and alertness scores [39]. Evidence for high‐fat interventions was also limited and inconsistent; cognitive and psychomotor outcomes showed considerable variability across studies [35, 41], highlighting the complexity and the need for more research approaches regarding specific macronutrient compositions.

### 4.1 Diet Quality

Beyond macronutrient composition and meal timing, thirteen studies examined components of diet quality. Of these, one study measured pro‐inflammatory biomarkers [64], and a small pool of observational studies considered the inflammatory potential of eating patterns using the Dietary Inflammatory Index (DII) (*n* 3) [56, 69, 70]. The findings collectively emphasise that diet quality, characterised by minimally processed foods, unsaturated fats, fruits, and vegetables, can play an important role in influencing a wide range of health outcomes for shift workers; from metabolic health and inflammatory markers [64, 70] to cognitive performance [66], mood [67], and sleep [69]. The evidence suggests that while poor dietary choices exacerbate the inherent health risks associated with shift work [59, 60, 62, 64, 65, 69, 70], nutritional strategies that focus on healthier eating patterns can offer significant benefits [64, 69].

To support shift worker health, nutritional interventions should emphasise improving overall diet quality through actionable and practical advice and/or initiatives that take into consideration any workplace and food accessibility constraints. For instance, specific advice to manage post‐prandial glucose levels and fatigue, while implementation of programmes to provide healthy meal options in the workplace have been shown to improve dietary intake among shift workers [67]. This demonstrates that organisational support can be a powerful tool for promoting better nutrition.

### 4.2 First Responder Populations

For emergency service workers and first responders, rotating shift patterns that routinely include night work are integral to the 24/7 operational function of these occupations. Given the dynamic and unpredictable nature of first responders' roles, alongside the suboptimal dietary intake reported [21], these groups may be more susceptible to experiencing health consequences.

The small number of studies (*n* 4, 9%) and variability in design illustrate the early stage of research investigating dietary interventions within first responder populations. This variability includes diverse intervention types, outcome measures, and varying durations, making direct comparisons and the drawing of firm conclusions challenging. Further controlled trials are warranted to explore how specific dietary strategies, tailored to the unique context of first responder occupations, can effectively mitigate the adverse metabolic and health effects that are associated with shift work. Moreover, the predominance of male participants and inconsistent reporting of shift work exposure highlight the need for more diverse samples and ecologically valid study designs that reflect real‐world operational schedules and physiological demands of their work. Addressing these gaps will be paramount for developing feasible, evidence‐based nutrition interventions that enhance health, performance, and resilience in shift working populations.

### 4.3 Strength of Evidence: Risk of Bias Assessment

The risk of bias assessment highlighted potential challenges of conducting research in shift working populations. These limitations highlight that current evidence should be interpreted with caution, particularly where findings are drawn from small, heterogeneous samples or studies lacking adequate control for confounding factors such as sleep duration, workload, or baseline diet quality. The predominance of crossover and observational designs limits causal inference, although these studies provide valuable ecological insight into real‐world behaviours. To strengthen the evidence base, future research should prioritise well‐controlled, adequately powered trials that incorporate objective measures of dietary intake, sleep, and metabolic health, alongside clear reporting of shift patterns and intervention fidelity. Improved methodological rigour and transparency will be essential to determine the true efficacy and feasibility of nutrition‐based interventions aimed at improving health and performance in shift‐working populations.

### 4.4 Evidence of Effectiveness

There was considerable heterogeneity in dietary strategies, intervention or exposure utilised, and measured outcomes across the included studies. This methodological variability has impacted how data could be analysed and interpreted. Therefore, studies based on similar interventions or outcomes were grouped to allow qualitative comparisons and identify trends or contradictions. Beyond the interventions and outcomes, the review also highlighted variations in study design, duration, and methods for measuring exposure and outcome. These methodological differences further contribute to heterogeneity and, due to impacts on reliability and validity, must be considered when interpreting results. The diverse nature of the included studies reduces their generalisability across all dietary contexts, health markers, or occupational groups.

### 4.5 Limitations and Gaps in the Evidence

The evidence base remains limited and methodologically diverse. The substantial heterogeneity of study designs, populations, and intervention protocols precluded meta‐analysis, thereby restricting the ability to draw strong, generalisable conclusions or develop precise quantitative recommendations. Furthermore, there is insufficient evidence within first responder populations to justify a dedicated systematic review, highlighting a significant research gap in groups who may be at greatest occupational risk.

The predominance of laboratory‐based, mechanistic studies within the evidence‐base may limit the ecological validity of some shift work research. Though highly valuable for their insights, due to the precise control over meal timing, composition, and sleep‐wake cycles; simulated protocols may not accurately replicate the operational realities of shift work, nor might ‘shift work naïve’ participants mirror the adaptive responses of experienced shift workers. In occupational environments, access to food and opportunities for rest are frequently constrained by workload and unpredictable demands [76, 77]. As such, the high adherence rates and physiological responses observed in laboratory conditions may not be directly transferable to free‐living shift workers. Addressing this limitation through ecologically valid, workplace‐based trials will be critical to ensure that future nutritional guidance is both evidence‐based and practically achievable.

High risk of bias across many included studies further constrains interpretability, reflecting common challenges such as small sample sizes, lack of randomisation, and incomplete control of confounding variables including sleep duration, physical activity, and baseline dietary habits. This reinforces the need for more standardised outcome measures and transparent reporting to better elucidate the complex relationships between nutrition, circadian disruption, and health in shift‐working populations. Strengthening the methodological quality and consistency of future research will be essential to generate reliable, actionable evidence capable of informing occupational health policy and practice.

### 4.6 Strengths and Limitations of This Systematic Review

This systematic review employed a robust methodology to ensure comprehensive and unbiased evidence synthesis. The search strategy was thorough, incorporating extensive database searches, retrieval of grey literature, and snowball searching from reference lists to minimise publication bias and identify the broadest possible range of relevant evidence. Furthermore, searches were re‐run prior to the final analysis to capture the most recent publications. Rigorous study selection and data extraction processes were implemented, with all included papers independently screened by two reviewers at both the title/abstract and full‐text stages. A third reviewer resolved any disagreements through discussion and consensus, and data extraction was also conducted independently by two reviewers, enhancing the reliability of the findings.

Given the observed heterogeneity in interventions, outcome measures, and reporting practices across the included studies, a meta‐analysis was deemed inappropriate. Instead, the review followed systematic review without meta‐analysis (SWiM) reporting guidelines, employing a qualitative synthesis to accurately depict overall trends, thus demonstrating a methodologically sound decision based on the nature of the available evidence.

This systematic review acknowledges several limitations that warrant consideration. While the inclusion of a pre‐defined search strategy enhances the credibility and reproducibility of the findings, not all occupational groups engaged in shift work were systematically searched, potentially resulting in the omission of relevant evidence. Grey literature searches were primarily conducted within UK‐based first responder organisations due to accessibility constraints. As grey literature is not consistently indexed across international contexts, this may have introduced geographical bias and limited the identification of relevant unpublished studies from other regions. To mitigate this, broader database searches, including ProQuest, were conducted without geographical restriction. However, the practical challenges of systematically accessing organisation‐specific grey literature across countries limit feasibility. Importantly, the grey literature identified represented a small proportion of included studies and did not substantively influence the overall findings, suggesting this limitation is unlikely to have altered the review conclusions. Nonetheless, some relevant international studies may have been missed.

A limitation of this review is that risk of bias assessment was conducted by a single reviewer. which may increase the potential for subjective interpretation. Although established assessment tools and predefined criteria were used to enhance consistency, the absence of independent duplicate assessment may reduce the robustness of bias evaluation. Future reviews would benefit from independent assessment by multiple reviewers to strengthen methodological rigour. Furthermore, some studies, or secondary analyses within them, were categorised as observational studies. While this was a necessary decision given the nature of the evidence, it means that the inherent limitations of observational designs, such as susceptibility to confounding and difficulties in establishing causality, are carried through into the review's synthesis.

Another notable limitation pertains to the scope of included interventions, as studies specifically investigating caffeine and hydration in shift‐working populations were explicitly excluded, due to another registered review that is ongoing (PROSPERO CRD42024509202).

Finally, although the literature search did not impose date restrictions, it is acknowledged that reporting guidelines for research studies, particularly for observational designs, have undergone significant evolution over time. Studies published prior to approximately 2010 might adhere to less stringent or different reporting standards. This historical context could subtly impact the comprehensiveness and transparency of their methodological descriptions, thereby influencing the consistency and ease of assessing their risk of bias and potentially affecting the overall interpretation of results.

### 4.7 Research and Practice Recommendations

To advance the understanding and application of nutritional interventions for shift workers, future research should prioritise the development of pragmatic, workplace‐based trials that are cognisant of the occupational realities of shift workers. A recent study in first responders identified that participants were motivated to improve their dietary habits, however unique occupational barriers were a challenge [78]. A participatory approach would ensure that recommendations are not only scientifically sound but also feasible and acceptable within the real‐world contexts of shift working occupations.

Additionally, this review has highlighted several key factors that could improve the validity and impact of nutritional intervention studies in shift workers. Specifically:

- Recruit diverse occupational groups, particularly under‐represented populations such as emergency service personnel.
- Incorporate objective measures of adherence (e.g., wearable technology, dietary tracking apps) to quantify real‐time compliance and behavioural patterns.
- Employ longitudinal designs to evaluate sustainability and long‐term health outcomes.
- Integrate composite indicators of diet quality such as the Dietary Inflammatory Index, Healthy Eating Index, or Mediterranean Diet Score.

Overall, the research suggests that reducing food intake during night‐time hours, and adoption of lower carbohydrate and higher protein intakes that simultaneously improve diet quality, hold promise for improving health‐related outcomes. However, evidence remains limited by short intervention durations and small sample sizes. While individual health benefits of TRE are evident, the effect on occupational performance (cognition and alertness) in highly demanding shift working occupations is unclear. Further research is required to addresses the complex interplay of health, cognitive function, and occupational performance in highly demanding, irregular‐shift professions. Longitudinal, ecologically valid studies are needed to confirm the sustainability and occupational feasibility of TRE and macronutrient protocols in real‐world shift‐working populations. Further studies should focus on practical, sustainable approaches that demonstrably enhance health *and* critical occupational performance in free‐living shift working populations, without introducing undue risks.

## 5 Conclusion

The review identified 43 studies addressing dietary interventions in shift‐working populations; however, only a small proportion (*n* 4, 9%) were conducted within first responder groups such as firefighters and police officers. The included studies exhibited considerable heterogeneity in study design, interventions, and outcomes, alongside a moderate to high risk of bias.

Overall, the evidence base is limited and heterogeneous, and there is currently no clear consensus on the most effective dietary approaches for shift workers. As such, the available evidence is insufficient to formulate definitive, evidence‐based dietary guidelines for this population. However, within the limited evidence, aligning eating patterns with circadian rhythms may confer health and performance benefits. Further high‐quality, pragmatic research addressing the limitations identified in this review is required to inform the development of robust nutritional guidelines for shift workers.

## Author Contributions

All authors made substantial contributions to the conception and design of the review, and to the interpretation of the findings. Alison Jayne Booker led the literature searches, screening, data extraction, analysis, and preparation of the manuscript. Rachel Gibson and Steven Powell contributed to the screening process through support with study inclusion and exclusion decisions. All authors contributed to the drafting and critical revision of the manuscript, provided intellectual guidance throughout the research process, and approved the final version of the manuscript prior to submission.

## Ethics Statement

As this study is a systematic review evaluating previously published, publicly available secondary literature, it did not directly involve human subjects or animal models. Consequently, formal institutional review board (IRB) ethical approval and participant informed consent were not required. All primary studies included in this review were assessed to ensure they were conducted under appropriate institutional ethical approvals and complied with the Declaration of Helsinki. The protocol for this systematic review was prospectively registered with PROSPERO (Registration Number: CRD42024509202).

## Conflicts of Interest

R.G. holds unpaid positions as Research Lead British Dietetic Research Programme, and Research Advisor Hercuwise Ltd.

## Supporting information


Supporting File

## Data Availability

The data that support the findings of this study are available from the corresponding author upon reasonable request.
