# Supplementary material for: A Systematic Review of Nutrition Interventions for Shift Workers, With Subgroup Analysis of First Responder Populations
Source: J Hum Nutr Diet. 2026 Oct 1;39(5):e70340. doi: 10.1111/jhn.70340 (PMC13629591; doi:10.1111/jhn.70340)
Supplement: Supplementary file 1 [file JHN-39-0-s001.docx]

**Contents**

Supplementary Table 1: Search Strategy and Search Terms

Supplementary Table 2: Full list of study characteristics by publication

Supplementary Table 3: Full list of intervention characteristics and primary outcomes by publication

Supplementary Table 4. Diet quality and dietary patterns and outcomes

Supplementary Table 5: Meal timing and outcomes

Supplementary Table 6: Shift work conditions - phase shift and circadian misalignment

Supplementary Table 7: Dietary Inflammatory Index (DII) and outcomes

Supplementary Table 8: First Responder studies and outcomes

Supplementary Table 9. ROBINS-I V2 Risk of Bias Tool for C-RCT

Supplementary Table 10. Newcastle-OTTOWA Risk of Bias Tool for Observational Studies

Supplementary Table 11. COCHRANE Risk of Bias Tool for RCT

**Supplementary Table 1: Search Strategy and Search Terms**

**Evidence of benefit: Dietary recommendations in emergency service and first responder shift workers**

### Search template:

| Concept 1  **Shift work** | AND | Concept 2  **Emergency Services** | AND | Concept 3  **Diet** |
| --- | --- | --- | --- | --- |
| Shiftwork* **OR** “shift work*” **OR** shift-work* **OR** “Night shift*” **OR** nightshift* **OR**  Shift* **OR** Night duty **OR** “Night work*” **OR** “night hour*” **OR** “evening work” **OR** work routine*  **OR** “split shift” **OR** split-shift **OR** “non day work” **OR** “extended shift” **OR** “extended work*” **OR** “long work” **OR** “long working” **OR** “non day work*” **OR** “non-day-work*” **OR** “long shift” **OR** “12-hour shift” **OR** “12 hour shift” **OR** “unsociable hour*” **OR** "atypical work hours"  **OR** rota **OR** roster*  **OR** circadian* **OR** “light dark cycle” **OR** “body clock” **OR** “chrono type” |  | **OR** First responder* **OR** emergency responder* **OR** responder **OR** 999  **OR** police officer* **OR** police*  **OR** officer* **OR** law enforcement **OR** Police service* **OR** Police force* **OR**  **OR** emergency service* **OR** “Blue light” service*  **OR** fire service* **OR** firefighter*  **OR** ambulance* **OR** paramedic* **OR** ambulance service* |  | **OR** Nutrition* **OR** Nutritional intake **OR** “chrono nutrition” **OR** “macro nutrient” **OR** macronutrient* **OR** fat **OR** lipid* **OR** protein **OR** carbohydrate* **OR** glucose **OR** “glycaemic index” **OR** nutrient*  **OR** Diet* **OR “**Dietary intake*” **OR “**Dietary recommendation*” **OR “**Dietary guidance”  **OR “**Food intake*” **OR** Food **OR** Food prep* **OR** Food record* **OR** food diary **OR** snack*  **OR “**Meal planning” **OR “**Meal prep***” OR** Meal* **OR** “Meal timing”* **OR** timing **OR** mealtime* **OR** “eating frequency” **OR** “meal frequency” **OR** “eating occasions” **OR** appetite |

*NB. Hydration and caffeine currently being reviewed in PROSPERO CRD42024509202*.

**Cochrane and PubMed**

Shiftwork OR “shift work” OR “Night shift” OR nightshift OR shift OR “Night duty” OR “Night work” OR “night hour” OR “evening work” OR “work routine*” OR “split shift” OR “non day work” OR “extended shift” OR “extended work” OR “long work” OR “long working” OR “non day work*” OR “long shift” OR “12 hour shift” OR “unsociable hour” OR "atypical work hours" OR rota OR roster

AND Diet OR “Dietary intake” OR “Dietary recommendation” OR “Dietary guidance” OR “Dietary guideline” OR Nutrition OR “Nutritional intake” OR “chrono nutrition” OR “macro nutrient” OR macronutrient OR fat OR protein OR carbohydrate OR glucose OR “glycaemic index” OR nutrient OR “Food intake” OR Food OR “Food prep” OR “Food record” OR “food diary” OR snack OR “Meal planning” OR Meal OR “Meal timing” OR mealtime OR “eating frequency” OR “meal frequency” OR “eating occasions” OR “Time restricted eating” OR “Time restricted feeding” OR “Intermittent fasting” OR “Nutrient timing” OR “Eating pattern”

AND “First responder” OR “emergency responder” OR responder OR 999 OR police officer OR police OR officer OR “law enforcement” OR “law enforcement officer” OR “Police service” OR “Police force” OR Cop OR “Patrol officer” OR “Police Department” OR “Sheriff Department” OR “Law and Order” OR “emergency service” OR “Blue light” service OR “Rescue service” OR “fire service” OR firefighter OR “fire officer” OR ambulance OR paramedic OR ambulance service

**SCOPUS / Web of Science**

Shiftwork OR "shift work" OR "Night shift" OR nightshift OR shift OR "Night duty" OR "Night work" OR "night hour" OR "evening work" AND Diet OR "Dietary intake" OR "Dietary recommendation" OR "Dietary guidance" OR "Dietary guideline" OR Nutrition OR "Nutritional intake" OR "chrono nutrition" OR "macro nutrient" OR macronutrient OR nutrient OR "Food intake" OR "Food record" OR "food diary" OR snack OR "Meal planning" OR Meal OR "Meal timing" OR mealtime OR "eating frequency" OR "meal frequency" OR "eating occasions" OR "Time restricted eating" OR "Time restricted feeding" OR "Intermittent fasting" OR "Nutrient timing" OR "Eating pattern"

AND

Diet* **OR “**Dietary intake*” **OR “**Dietary recommendation*” **OR “**Dietary guidance” **OR** **“**Dietary guideline*” **OR** Nutrition* **OR “**Nutritional intake” **OR** “chrono nutrition” **OR** “macro nutrient” **OR** macronutrient* **OR** fat **OR** protein **OR** carbohydrate* **OR** glucose **OR** “glycaemic index” **OR** nutrient* **OR “**Food intake*” **OR** Food **OR “**Food prep*” **OR “**Food record*” **OR** “food diary” **OR** snack* **OR “**Meal planning” **OR** Meal* **OR** “Meal timing”* **OR** mealtime* **OR** “eating frequency” **OR** “meal frequency” **OR** “eating occasions” **OR “**Time restricted eating” **OR “**Time restricted feeding” **OR** “Intermittent fasting” **OR** “Nutrient timing” **OR** “Eating pattern*”

AND

**“**First responder*” **OR “**emergency responder*” **OR** responder **OR** 999 **OR** police officer* **OR** police* **OR** officer* **OR “**law enforcement” **OR “**law enforcement officer*” **OR “**Police service*” **OR “**Police force*” **OR** Cop* **OR** “Patrol officer*” **OR** “Police Department*” **OR** “Sheriff* Department*” **OR** “Law and Order” **OR** “emergency service”* **OR** “Blue light” service* **OR** “Rescue service*”**OR “**fire service”* **OR** firefighter* **OR “**fire officer”* **OR** ambulance* **OR** paramedic* **OR** ambulance service*

**Supplementary Table 2: Full list of study characteristics by publication**

| **Study ID** | **Study Design** | **Country** | **Occupation** | **Shift Type** | **Number participants** | **Sex, %** | **Age, years (SD)** | **BMI, kg/m2 (SD)** | **Ethnicity, % white** |
| --- | --- | --- | --- | --- | --- | --- | --- | --- | --- |
| Aarrebo Jensen et al., (2020) | C-RCT | Denmark | Nursing and Healthcare | Mixed | 10 | 100% (f) | 51 (8.1) | 29.9 (6.1) | NR |
| Al-Naimi et al., (2004) | C-RCT | UK | Students | Simulated | 8 | 100% (m) | 20-33 | 20-25 | NR |
| Binder-Mendl et al., (2022) | C-RCT | Austria | Industrial Worker | NR | 19 | 100% (m) | 42.7 (6.6) | Not reported | NR |
| Bonham et al., (2020) | C-RCT | Australia | Simulation participants | Simulated | 16 | 100% (m) | 25 (9) | 35.1 (10.6) | NR |
| Centofanti et al., (2018) | C-RCT | Australia | Simulation participants | Simulated | 31 | 58% (f)  42% (m) | 24.3 (3.4) | 22.2 (3) | NR |
| Chellappa et al., (2021) | RCT | USA | Simulation participants | Simulated | 20 | 60% (m) 40% (f) | 26.6 (4.2) | 22.7(2.1) | NR |
| Cunha et al.,(2020) | C-RCT | Brazil | Nursing and Healthcare | Fixed | 14 | 100% (m) | 40.9 (8.9) | 29.1 (5.3) | NR |
| de Rijk et al., (2021) | Observational Study | Netherlands | Nursing and Healthcare | NR | 118 | 100% (f) | 44.2 (10.8) | Not reported | NR |
| Grant et al., (2017a) | C-RCT | Australia | Simulation participants | Simulated | 10 | 100% (m) | 25.2 (6.4) | 22.7 (1.9) | NR |
| Grant et al., (2017b) | C-RCT | Australia | Simulation participants | Simulated | 11 | 100% (m) | 25 (5.4) | 22.25 (1.75) | 27% |
| Gupta et al., (2021) | C-RCT | Australia | Simulation participants | Simulated | 61 | 59% (m) 39% (f) | 24.5 (2.3) | 23.4 (2.3) | NR |
| Gupta et al., (2017) | C-RCT | Australia | Simulation participants | Simulated | 10 | 100% (m) | 24.7 (5.55) | 22.72 (1.28) | NR |
| Gupta et al., (2019a) | RCT | Australia | Simulation participants | Simulated | 44 | 90% (m) 10% (f) | 25 (4.9) | 23.8 (2.6) | NR |
| Gupta et al., (2019b) | RCT | Australia | Simulation participants | Simulated | 44 | 90% (m) 10% (f) | 25 (4.9) | 23.8 (2.6) | NR |
| Hampton et al., (1996) | C-RCT | UK | Students | Simulated | 9 | 66.7% (m) 33.3% (f) | 22 (1.75) | Not reported | NR |
| HolmbÃ¤ck et al., (2003) | C-RCT | Sweden | Simulation participants | Simulated | 7 | 100% (m) | 32 (2.25) | 23.8 (1.68) | NR |
| Jung et al., (2020) | Observational | South Korea | Nursing and Healthcare | NR | 1638 | 100% (f) | NR | NR | 100% IC4 |
| Khorasaniha et al., (2020) | Observational | Iran | Factory worker | Rotating | 257 | 100% (m) | 37 (2.88) | 26.3 (2.81) | 100% IC6 (MENA) |
| Khorasaniha et al., (2022) | Observational | Iran | Factory worker | Rotating | 257 | 100% (m) | 37 (2.88) | Not reported | NR |
| Knutsson et al., (2002) | C-RCT | Sweden | Nursing and Healthcare | Mixed | 11 | 100% (f) | 42 (2.5) | 25 (2.4) | NR |
| Kräuchi et al., (1990) | Observational | Switzerland | Nursing and Healthcare | Fixed | 28 | 64% (m) 36% (f) | 29.2 (6.8) | Not reported | NR |
| Kulak et al., (2022) | Observational | Turkey | Nursing and Healthcare | Rotating | 100 | 58% (f)  42% (m) | 37.8 (6.07) | 27.6 (3.56) | NR |
| Leedo et al., (2017) | C-RCT | Denmark | Nursing and Healthcare | NR | 16 | NR | 40.9 (10.5) | 24.8 (3.9) | NR |
| Leung et al., (2021) | C-RCT | Australia | Mixed | Mixed | 19 | 68.4% (f) 31.6% (m) | 41 (10) | 30.7 (5.7) | NR |
| Love et al., (2005) | C-RCT | Canada | Factory worker | Rotating | 6 | 66.7% (m) 33.3% (f) | 24 (6.25) | 24 (n.d) | NR |
| Lowden et al., (2004) | C-RCT | Sweden | Simulation participants | Simulated | 8 | 100% (m) | 31.4 (1.9) | 23.7 (0.9) | NR |
| Lowden et al., (2001) | Observational | Sweden | Simulation participants | Simulated | 8 | 100% (m) | 31.4 (1.9) | 23.7 (0.9) | NR |
| Makowski et al., (2022) | RCT | USA | Nursing and Healthcare | Rotating | 61 | 50.8% (f) 49.2% (m) | 29.7 (2.2) | 23 (3.3) | NR |
| Manoogian et al., (2022) | RCT | USA | Firefighter | Rotating | 137 | 91.2% (m) 8.8% (f) | 40.36 (90.4) | 29.68 (94.21) | 60% |
| Molzof et al., (2022) | Observational | USA | Nursing and Healthcare | Rotating | 18 | 100% (f) | 30 (1) | 27.2 (5.6) | 100% |
| Molzof et al., (2017) | Observational | USA | Nursing and Healthcare | Rotating | 17 | 100% (f) | 32.4 (10.8) | 28.1 (7.1) | 88.2% |
| Morris et al., (2016) | RCT | USA | Nursing and Healthcare | Simulated | 9 | 67% (f) 33% (m) | 34 (8) | 24.2 (3.4) | NR |
| Nehme et al., (2014) | RCT | Brazil | Security Guards | Fixed | 51 | 100% (m) | 30.8 (5.5) | 28.9 (4.2) | NR |
| O'Brien et al., (2020) | Observational | Ireland | Mixed | NR | 1080 | 55.8% (m) 44.2% (f) | 40.4 (nd) | Not reported | NR |
| Oriyama & Yamashita (2021) | C-RCT | Japan | Simulation participants | Simulated | 15 | 100% (f) | 21.7 (0.5) | 19.81 (2.45) | NR |
| Park et al., (2023) | Observational | South Korea | Nursing and Healthcare | Rotating | 128 | 6.3% (m) 93.8% (f) | 26.9 (5.69) | 21 (3.06) | NR |
| Paz & Berry (1997) | C-RCT | Isreal | Nursing and Healthcare | Rotating | 21 | 57% (f) 43% (m) | 31.26 (5.85) | Not reported | NR |
| Rodrigues et al., (2022) | Observational | Brazil | Nursing and Healthcare | Fixed | 39 | 100% female | 38.2 (nd) | 29.9 (nd) | NR |
| Silva et al., (2020) | C-RCT | Brazil | Nursing and Healthcare | NR | 16 | 100% (m) | 41 (8.8) | 28.8 (5) | NR |
| Smith-Ryan et al., (2019) | RCT | USA | Nursing and Healthcare | Rotating | 41 | 100% (f) | 30.5 (7.7) | 27.4 (5.4) | 94% |
| Teixeira et al., (2022) | C-RCT | Brazil | Police Officer | Fixed | 10 | 100% (m) | 38.8 (4) | 25.9 (1.9) | NR |
| Wirth et al., (2014) | Observational | USA | Police Officer | NR | 447 | 75% (m) 25% (f) | 42.4 (8.5) | 29.3 (4.8) | 79% |
| Wirth et al., (2022) | Observational | USA | Police Officer | NR | 240 | 74% (m) 26% (f) | 41.5 (6.7) | 29.3 (4.8) | 77% |
| **Footnotes:** NR: Not Reported; IC: Identity Code; IC1: White N. European, IC2: White S. European, IC3: Black, IC4: Asian – Indian subcontinent, IC5: SE Asia, Chinese, Japanese, Korean, IC6: Arab or North African | | | | | | | | | |

**Supplementary Table 3: Full list of intervention characteristics and primary outcomes by publication**

| **Study ID** | **Study Design** | **Intervention** | **Intervention duration*** | **Primary outcomes** | **Tool** | **Measurement** | **Effect type: within/bet-ween** | **Occupation** |  |
| --- | --- | --- | --- | --- | --- | --- | --- | --- | --- |
| Aarrebo Jensen et al., (2020) | C-RCT | Sugar intake | 4-days | Post-prandial glucose | Food log | Blood glucose | Within-person (within-group) | Nursing and Healthcare |  |
| Al-Naimi et al., (2004) | C-RCT | Meal timing | 2-days | Metabolic profiles | Serum | Total AUC | Within-person (within-group) | Students |  |
| Binder-Mendl et al., (2022) | C-RCT | Nutritional counselling | 1-year | Metabolic profiles | Serum | Values per parameter | Both | Industrial Worker |  |
|  |  |  |  | Nutrient intake | Food record | Nutritional analysis |  |  |  |
| Bonham et al., (2020) | C-RCT | Macronutrient composition | 2-days | Metabolic profiles | Serum | iAUC | Within-person (within-group) | Simulation participants |  |
| Centofanti et al., (2018) | C-RCT | Meal timing and quantity | 3-days | Metabolic profiles | Serum | Total AUC | Between-person | Simulation participants |  |
| Chellappa et al., (2021) | RCT | TRE | 4-days | Circadian misalignment | Serum | iAUC | Parallel-group(between-subjects) | Simulation participants |  |
|  |  |  |  | Metabolic profiles | Saliva | Melatonin |  |  |  |
| Cunha et al.,(2020) | C-RCT | Macronutrient composition | 2-days | Metabolic profiles | Serum | iAUC | Within-person (within-group) | Nursing and Healthcare |  |
| de Rijk et al., (2021) | Observational Study | Meal frequency | 3-days | Alertness | Cognitive test | Subjective | Both | Nursing and Healthcare |  |
|  |  |  |  | GI complaints | Questionnaire |  |  |  |  |
| Grant et al., (2017a) | C-RCT | TRE | 6-days | Cognition | Cognitive test | Subjective | Between-person | Simulation participants |  |
|  |  |  |  | GI complaints | Questionnaire |  |  |  |  |
| Grant et al., (2017b) | C-RCT | TRE | 6-days | Metabolic profiles | Serum | iAUC | Between-subjects (parallel) | Simulation participants |  |
| Gupta et al., (2021) | C-RCT | TRE | 7-days | Driving performance | Simulations | Test scores | Between-person | Simulation participants |  |
| Gupta et al., (2017) | C-RCT | TRE | 7-days | Driving performance | Simulations | Test scores | Between-person | Simulation participants |  |
| Gupta et al., (2019a) | RCT | Meal timing and quantity | 7-days | Driving performance | Simulations | Mixed-effects ANOVA | Between-person | Simulation participants |  |
|  |  |  |  | Cognition | Cognitive tasks |  |  |  |  |
|  |  |  |  | Sleepiness | Subjective |  |  |  |  |
| Gupta et al., (2019b) | RCT | Meal timing and quantity | 7-days | Hunger/fullness | Subjective | Mixed-effects ANOVA | Between-person | Simulation participants |  |
|  |  |  |  | Sleepiness |  |  |  |  |  |
|  |  |  |  | GI complaints |  |  |  |  |  |
| Hampton et al., (1996) | C-RCT | Shift work condition | 15-days | Metabolic profiles | Serum | Total AUC | Within-person (within-group) | Students |  |
| Holmbäck et al., (2003) | C-RCT | Macronutrient composition | 2 x 7-days | Metabolic profiles | Serum | ANOVA  (3 factor) | Within-person (within-subjects) | Simulation participants |  |
| Jung et al., (2020) | Observational | Diet quality and dietary behaviours | NR | MetS Prevalence | Anthropometric + Serum | BCM | Between-person | Nursing and Healthcare |  |
| Khorasaniha et al., (2020) | Observational | Diet quality | NR | Proinflammatory cytokines | Serum | Inflammatory biomarkers | Between-person | Factory worker |  |
| Khorasaniha et al., (2022) | Observational | Diet quality | SPoC** | Sleep disturbances | Subjective | Epworth scale | Between-person | Factory worker |  |
| Knutsson et al., (2002) | C-RCT | Meal timing and quantity | 1-week | Metabolic profiles | Serum | Total AUC | Within-person | Nursing and Healthcare |  |
| Kräuchi et al., (1990) | Observational | Diet quality and dietary patterns | 4-weeks | Cognition + sleep | Subjective | Likert scale | Within-person | Nursing and Healthcare |  |
| Kulak et al., (2022) | Observational | Shift work condition | 2-months | Dietary intake | Food log | Self-report |  | Nursing and Healthcare |  |
|  |  |  |  | Cognition | Montreal Assessment | Test score | Between-person |  |  |
|  |  |  |  | Anthropometrics | BMI | Objective |  |  |  |
| Leedo et al., (2017) | C-RCT | Diet quality / workplace meal provision | 9-weeks | Reaction time | Performance test | Test score | Within-person | Nursing and Healthcare |  |
|  |  |  |  | Mood | Questionnaire | Subjective |  |  |  |
|  |  |  |  | Diet and hydration | Food log |  |  |  |  |
| Leung et al., (2021) | C-RCT | TRE | 2-months | Metabolic profiles | Serum | iAUC | Within-person | Mixed |  |
| Love et al., (2005) | C-RCT | Macronutrient composition | 2-weeks | Cognition | Cognitive task | PASAT Score | Within-person | Factory worker |  |
|  |  |  |  | Sleep | Sleep scale |  |  |  |  |
| Lowden et al., (2004) | C-RCT | Macronutrient composition | 2 x 1-week | Cognition | Cognitive tests | ANOVA | Within-person | Simulation participants |  |
| Lowden et al., (2001) | Observational | Macronutrient composition | NR | Mood + hunger | Subjective scale | ANOVA | Between-person | Simulation participants |  |
| Makowski et al., (2022) | RCT | Macronutrient composition | 1-week | Neuro-behavioural performance | Cognitive tests | Linear mixed models | Within-person | Nursing and Healthcare |  |
| Manoogian et al., (2022) | RCT | TRE | 12-weeks | Metabolic profiles | Serum | Mixed ANOVA | Between-person | Firefighter |  |
|  |  |  |  | TRE feasibility | Interviews | Qualitative |  |  |  |
| Molzof et al., (2022) | Observational | Meal timing and quantity | 9-days | Metabolic profiles | Serum | Mixed ANOVA | Between-person | Nursing and Healthcare |  |
| Molzof et al., (2017) | Observational | Diet quality / inflammation | 9-days | DII | Serum | Metabolic parameters | Between-person | Nursing and Healthcare |  |
|  |  |  |  |  | Food diary | DII Score |  |  |  |
| Morris et al., (2016) | RCT | Shift work /  phase shift | 2 x 3-days | Metabolic profiles | Serum | AUC | Within-person | Nursing and Healthcare |  |
| Nehme et al., (2014) | RCT | Macronutrient composition | 3-weeks | Sleep | Actigraphy | ANOVA  (2 factor) | Between-person | Security Guards |  |
| O'Brien et al., (2020) | Observational | Diet quality | 6-weeks | Overweight / obesity | Anthropometrics | BMI | Between-person | Mixed |  |
| Oriyama & Yamashita (2021) | C-RCT | TRE | 2 x 2-days | Cognition | Cognitive test | Test result | Within-person | Simulation participants |  |
|  |  |  |  | Sleep | Scale | Subjective |  |  |  |
| Park et al., (2023) | Observational | Meal timing and quantity | 1-day | Sleep | Total Sleep Time / Quality | Subjective | Between-person | Nursing and Healthcare |  |
| Paz & Berry (1997) | C-RCT | Macronutrient composition | 3 x 3-days | Cognition | Cognitive tests | Repeated measures ANOVA | Between-person | Nursing and Healthcare |  |
|  |  |  |  | Sleep | Actigraphy |  |  |  |  |
|  |  |  |  | Metabolic profiles | Serum |  |  |  |  |
| Rodrigues et al., (2022) | Observational | Diet quality and dietary patterns | 2-days | Sleep quality | Self-report | Pittsburgh sleep index | Between-person | Nursing and Healthcare |  |
| Silva et al., (2020) | C-RCT | Macronutrient composition | 7-days | Appetite + satiety | Subjective scale | ANOVA  (3 factor) | Within-person | Nursing and Healthcare |  |
| Smith-Ryan et al., (2019) | RCT | Probiotic/prebiotic blend | 6-weeks | Body composition | Visceral Adipose Tissue | ANOVA | Between-person | Nursing and Healthcare |  |
|  |  |  |  | Cholesterol | Serum |  |  |  |  |
|  |  |  |  | Mood | Surveys |  |  |  |  |
|  |  |  |  | Fatigue |  |  |  |  |  |
|  |  |  |  | Exercise performance | VO2 Max |  |  |  |  |
| Teixeira et al., (2022) | C-RCT | TRE | 3 x 2-days | Metabolic profiles | Serum | ANOVA | Within-person | Police Officer |  |
|  |  |  |  | Next day eating | Food log |  |  |  |  |
| Wirth et al., (2014) | Observational | DII | SPoC + follow-up over 5-years | Metabolic profiles | MetS markers | Linear models | Between-person | Police Officer |  |
|  |  |  |  | Inflammatory markers |  |  |  |  |  |
| Wirth et al., (2022) | Observational | DII | SPoC + follow-up over 5-years | Sleep quality | Objective + subjective measures | ANOVA | Between-person | Police Officer |  |
| **Footnotes:** *Data collection duration does not include wash-out periods, preparatory phases, or similar; **SPoC: Single Point of Collection; iAUC: incremental Area Under Curve; MetS: Metabolic Syndrome; BCM: Body Composition Measures; PASAT: Paced Auditory Serial Addition Test; DII: Dietary Inflammatory Index; NR: Not Reported | | | | | | | | | |

|  | **Supplementary Table 4. Diet quality and dietary patterns and outcomes** | | | | | | | | | | | | |
| --- | --- | --- | --- | --- | --- | --- | --- | --- | --- | --- | --- | --- | --- |
| **Study** | | **Design** | **Simu-lated** | **Duration of participant involvement** | **Intervention / exposure** | **Measure of exposure** | **Primary outcomes** | **Measurement of Outcome** | **Effects on / association with primary outcome** | | | **P-value** |  |
| Arrebo et al., 2020 [1] | | C-RCT | N | 4-days | Sugar intake | Food log | Post-prandial glucose | Blood glucose monitor |  | | | 0.0086 |  |
| de Rijk et al., 2021 [8] | | Obs | N | 3-days | Meal frequency Energy  Intake | 24-hour dietary recall | Alertness | Psychomotor vigilance task |  | | | 0.03 |  |
|  |  |  |  |  |  |  |  | Subjective Alertness |  | | | 0.291 |  |
|  |  |  |  |  |  |  | Gastro-intestinal complaints | Questionnaire |  | | | 0.493 |  |
| Jung et al., 2020 [17] | | Obs | N | >2-years | Eating speed | FFQ* + Survey | Prevalence  of MetS | MetS risk  factors: Abdominal obesity Serum |  | | | 0.002 |  |
|  |  |  |  |  | Calorie distribution |  |  |  |  | | | 0.001 |  |
|  |  |  |  |  | Alcohol consumption |  |  |  |  | | | 0.616 |  |
|  |  |  |  |  | Black coffee consumption |  |  |  |  | | | 0.507 |  |
|  |  |  |  |  | Carbonated drink intake |  |  |  |  | | | 0.002 |  |
| Khorasaniha et al., 2020 [18] | | Obs | N | NR | Diet quality / Diet pattern | FFQ | Pro-inflammatory cytokines | Serum | **  VEG^a^ | LFE^b^ | TRS^c^ | ^a^0.001 ^b^0.34 ^c^0.01 |  |
|  |  |  |  |  |  |  |  |  |  |  |  |  |  |
| Khorasaniha et al., 2022 [19] | | Obs | N | Single point of collection | Diet quality / Diet pattern | FFQ | Sleep disturbance | Epworth Sleepiness Scale | ***  Classic^a^ | Modern^b^ | Low veg^c^ | ^a^0.15 ^b^0.42 ^c^0.03 |  |
|  |  |  |  |  |  |  |  |  |  |  |  |  |  |
| Kräuchi H et al., 1990 [21] | | Obs | N | 4-weeks | Sugar intake | FFQ | Fatigue | 5-point Likert scale |  | | | <0.05 |  |
|  |  |  |  |  | Caffeine consumption |  |  |  |  | | | <0.05 |  |
| Leedo et al., 2017 [23] | | C-RCT | N | 9-weeks | Healthy meal provision in the workplace | 4-day dietary record | Reaction time | Attentional Performance |  | | | 0.581 |  |
|  |  |  |  |  |  |  | Mood | Questionnaire |  | | | 0.017 |  |
|  |  |  |  |  |  |  | Fluid intake | Food diary |  | | | <0.0001 |  |
|  |  |  |  |  |  |  | Total energy |  |  | | | 0.177 |  |
|  |  |  |  |  |  |  | Energy saturated fat |  |  | | | 0.003 |  |
|  |  |  |  |  |  |  | Dietary fibre |  |  | | | 0.031 |  |
| O'Brien et al., 2020 [34] | | Obs | N | 6-weeks | Consumption of fried foods | Questionnaire | Overweight and obesity | BMI |  | | | 0.001 |  |
|  |  |  |  |  | Fruit and veg |  |  |  |  | | | NR |  |
|  |  |  |  |  | Dairy |  |  |  |  | | | 0.359 |  |
|  |  |  |  |  | Alcohol |  |  |  |  | | | 0.035 |  |
| Rodrigues et al., 2022 [38] | | Obs | N | 2-days | Animal-based protein intake | Food diary | Sleep quality | Pittsburgh Sleep Index |  | | | NR |  |
|  |  |  |  |  | Fibre intake |  |  |  |  | | | NR |  |
| Smith-Ryan et al., 2019 [40] | | RCT | N | 6-weeks | Probiotic Prebiotic blend | Supplement compliance | Body composition | Visceral Adipose Tissue |  | | | 0.780 |  |
|  |  |  |  |  |  |  | Cholesterol | Serum |  | | | 0.623 |  |
|  |  |  |  |  |  |  | Mood | Surveys |  | | | 0.621 |  |
|  |  |  |  |  |  |  | Fatigue |  |  |  |  | 0.773 |  |
|  |  |  |  |  |  |  | Exercise performance | VO2 Max |  | | | 0.868 |  |
| **Footnotes:** Effect estimates include both within-group and between-group comparisons as reported in the original studies; this is specified for each outcome where applicable. *FFQ: Food Frequency Questionnaire. **VEG Dietary Pattern: characterised by green, yellow, and cruciferous vegetables, LFE Dietary Pattern: characterised by liquid oils, mayonnaise, fast food, and eggs; TRS Dietary Pattern: characterised by tea, coffee, refined grains, and spice. ***Classic (Iranian) characterised by high-protein animal foods, fruit, and vegetables; Modern (Iranian) characterised by high amounts of refined grains, solid oils, and caffeine drinks; High Protein Low Fruit & Veg characterised by animal proteins and solid oils with low amounts of fruit and vegetables. | | | | | | | | | | | | |  |
|  | | | | | | | | | | | | | |

**Supplementary Table 5: Meal timing and outcomes**

| **Study** | | **Study type** | **Simulated shiftwork** | **Timing Schedule** | **Primary outcomes** | | **Method of measurement** | | **Description** | | **Effect on primary outcome(s)** | | | | **Mean difference** | ***P* value** |
| --- | --- | --- | --- | --- | --- | --- | --- | --- | --- | --- | --- | --- | --- | --- | --- | --- |
| Al-Naimi et al., 2004 [2] | | C-RCT | Y | 3-hourly from 1900 | Glucose | | Serum | | Total AUC | |  | | | | -2.8 mmol/l.h | 0.05* |
|  |  |  |  |  | Insulin | |  |  |  |  |  | | | | +106 pmol/l.h | <0.01 |
|  |  |  |  |  | Triacylglycerol | |  |  |  |  |  | | | | +0.3 mmol/l.h | <0.05 |
|  |  |  |  |  | Non-esterified fatty acids | |  |  |  |  |  | | | | +0.3 mmol/l.h | >0.05 |
| Centofanti, S et al., 2018 [5] | | C-RCT | Y | 00:00-00:30 | Big snack (2100 KJ) | | Postprandial glucose | | Total AUC | |  | | | | +1.0 mmol/L | 0.001 |
|  |  |  |  |  | Small snack (840 KJ) | |  |  |  |  |  | | | | -0.2 mmol/L | >0.05 |
| Gupta at al., 2019a [13] | | C-RCT | Y | 00:30 | **  MN (30% daily Kcal)  SN (10% daily Kcal)  NE (0% daily Kcal) | | Driving performance | | Driving simulator | | MN^a^ | | SN^b^ | NE^c^ | - | ^a^<0.001  ^b^<0.001  ^c^<0.001 |
|  |  |  |  |  |  |  | Cognitive performance | | Reaction Time | |  | |  |  |  | ^a^<0.05  ^b^PC***  ^c^<0.05 |
|  |  |  |  |  |  |  |  |  | Memory Task | |  | |  |  |  | ^a^<0.001  ^b^PC  ^c^<0.001 |
|  |  |  |  |  |  |  | Sleepiness | | Subjective scale | |  | |  |  |  | ^a^<0.001  ^b^PC  ^c^PC |
| Gupta at al., 2019b [14] | | C-RCT | Y | 00:30 | MN (30% daily Kcal)  SN (10% daily Kcal)  NE (0% daily Kcal) | | Hunger | | Mixed-effects ANOVA | | MN^a^ | | SN^b^ | NE^c^ | - | ^a^<0.001  ^b^<0.001  ^c^<0.001 |
|  |  |  |  |  |  |  | Fullness | |  |  |  | |  |  |  | ^a^<0.001  ^b^<0.001  ^c^<0.001 |
|  |  |  |  |  |  |  | Sleepiness | |  |  |  | |  |  |  | NR |
|  |  |  |  |  |  |  | GI Discomfort | |  |  |  | |  |  |  | NR |
| Hampton et al., 1996 [15] | | C-RCT | Y | Phase shift | | Glucose | | Serum | | Total AUC | |  | | | 1.79 mmol/l.min | <0.02 |
|  |  |  |  |  |  | Insulin | |  |  |  |  |  | | | 58.98 pmol/l.min | <0.01 |
|  |  |  |  |  |  | Postprandial Triacylglycerol | |  |  |  |  |  | | | NR | <0.05 |
|  |  |  |  |  |  | Non-esterified fatty acids | |  |  |  |  |  | | | 42.5 mmol/l.min | <0.005 |
|  |  |  |  |  |  | GI hormones | |  |  |  |  |  | | | 3.88 nmol/l.min | NR |
| Knutsson et al., 2002 [20] | C-RCT | | N | 23:30 vs  03:30  Identical meals:  1860 KJ | Postprandial glucose | | Serum | | Total AUC | |  | | | | - | NR |
|  |  |  |  |  | Postprandial insulin | |  |  |  |  |  | | | |  | 0.03 |
|  |  |  |  |  | Postprandial triglycerides | |  |  |  |  |  | | | |  | NR |
| Molzof et al., 2022 [30] | Obs | | N | 50% food intake during nighttime | Insulin | | Serum | | 24hr values | |  | | | | 29.7 ± 5.7 mU/L | <0.0001 |
|  |  |  |  |  | Leptin | |  |  |  |  |  | | | | 23.1 ± 3.1 ng/ml | <0.0001 |
| Molzof et al., 2017 [31] | Obs | | N | 2100-0600 window | Dietary inflammatory index (DII) | | Food diary | | DII Score | |  | | | | - | 0.75 |
|  |  |  |  |  |  |  | Serum | | Metabolic parameters | |  | | | |  | NR |
| Park et al., 2023 [36] | Obs | | N | Meal-to-sleep interval 1.43 ± 1.18hrs | Post shift/pre-sleep meal  Self-selected | | Sleep Time | | Subjective scale | | **** | | | | -0.37 | 0.002 |
|  |  |  |  |  |  |  | Sleep Quality | |  |  |  | | | | -0.23 | 0.048 |
| **Footnotes:** *Statistically significant only after first meal. ** MN: Meal at Night, SN: Snack at Night, NEN: No Eating at Night. *** Statistically significant pairwise comparisons. **** Time to Sleep (TTS) as continuous variable: the shorter the TTS, the more statistically significant the outcomes. | | | | | | | | | | | | | | | | |

**Supplementary Table 6: Shift work conditions - phase shift and circadian misalignment**

| **Study** | **Study type** | **Simulated shiftwork** | **Shiftwork Schedule** | **Primary outcomes** | **Method of measurement** | **Description** | **Effect on primary outcome(s)** | **Mean difference** | ***P* value** |
| --- | --- | --- | --- | --- | --- | --- | --- | --- | --- |
| Kulak and Yesil., 2022 [22] | Obs | N | Fixed & Rotational | Anthropometrics | Objective | BMI |  | +1.0 mmol/L | 0.036 |
|  |  |  |  | Eating speed | Food diary | Self-report |  | - | 0.003 |
|  |  |  |  | Healthy snack choice |  |  |  | - | <0.05 |
|  |  |  |  | Energy intake |  |  |  | - | 0.07 |
|  |  |  |  | Cognitive functions | Screening | Montreal Assessment |  | - | 0.08 |
| Morris et al., 2016 [32] | RCT | Y | Phase shift | Postprandial glucose | Serum | AUC |  | 6.5% | 0.0041 |
|  |  |  |  | Postprandial insulin |  |  |  | 18% | 0.011 |

**Supplementary Table 7: Dietary Inflammatory Index (DII) and outcomes**

| **Study** | **Study type** | **Simulated shiftwork** | **DII Scores** | **Primary outcomes** | **Method of measurement** | **Description** | **Effect on primary outcome(s)** | **Mean difference** | ***P* value** |
| --- | --- | --- | --- | --- | --- | --- | --- | --- | --- |
| Wirth et al., 2014 [42] | Obs | N | High Vs Low Quartiles | Glucose intolerance | Serum | MetS markers |  | - | OR**: 2.03 (95%CI=1.08–3.82) |
|  |  |  |  | Insulin |  |  |  |  | 0.10 |
|  |  |  |  | Triacylglycerides |  |  |  |  | NR |
|  |  |  |  | Leptin |  |  |  |  | NR |
|  |  |  |  | CRP* |  |  |  |  | OR: 2.17 (95%CI=1.19–3.95) |
| Wirth et al., 2022 [43] | Obs | N | High Vs Low Quartiles | WASO*** | Objective measures | Sleep quality |  | - | 0.02 |
|  |  |  |  | Sleep latency |  |  |  |  | <0.01 |
|  |  |  |  | Sleep efficiency |  |  |  |  | 0.12 |
|  |  |  |  | PSQI**** | Subjective measures |  |  |  | 0.01 |
| **Footnotes:** *CRP: C-Reactive Protein, **OR: Odds Ratio, *** Wake-After-Sleep-Onset, ****PSQI: Pittsburgh Sleep Quality Index, | | | | | | | | | |

**Supplementary Table 8: First Responder studies and outcomes**

| **Study** | **Study type** | **Service** | **Intervent-ion detail** | **Primary outcomes** | **Method of measurement** | **Description** | **Effect on primary outcome(s)** | **Mean difference** | ***P* value** |
| --- | --- | --- | --- | --- | --- | --- | --- | --- | --- |
| Manoogian et al., 2022 [29] | RCT | Fire | Reduced eating window to 10hrs | TRE Feasibility | Questionnaires / interviews | Eating window |  | 95% CI 10.7–11.5 h | 3.29 × 10⁻¹⁷ |
|  |  |  |  | Fasting glucose | Serum | Fasting levels |  | 95% CI -0.41 to -0.01 mmol/L | 0.043 |
|  |  |  |  | VLDL Cholesterol Particle size |  |  |  | -1.34 (-2.20 - -0.49) | 0.003 |
| Teixeira et al., 2022 [41] | C-RCT | Police | NEN v Meal at 02:00 | Next day eating | Food diaries | Sugar intake |  | 19.1g (95% CI, -36.2 to -2.1) | <0.05 |
|  |  |  |  | Post-prandial glucose | Serum | iAUC |  | - | <0.05 |
| Wirth et al., 2014 [42] | Obs | Police | DII: High Vs Low Quartiles | Glucose intolerance | Serum | MetS markers |  | - | OR**: 2.03 (95%CI=1.08–3.82) |
|  |  |  |  | Insulin |  |  |  |  | 0.10 |
|  |  |  |  | Triacylglycerides |  |  |  |  | NR |
|  |  |  |  | Leptin |  |  |  |  | NR |
|  |  |  |  | CRP* |  |  |  |  | OR: 2.17 (95%CI=1.19–3.95) |
| Wirth et al., 2014 [42] | Obs | Police | DII: High Vs Low Quartiles | WASO*** | Objective measures | Sleep quality |  | - | 0.02 |
|  |  |  |  | Sleep latency |  |  |  |  | <0.01 |
|  |  |  |  | Sleep efficiency |  |  |  |  | 0.12 |
|  |  |  |  | PSQI**** | Subjective measures |  |  |  | 0.01 |
| **Footnotes:** *CRP: C-Reactive Protein, **OR: Odds Ratio, *** Wake-After-Sleep-Onset, ****PSQI: Pittsburgh Sleep Quality Index, | | | | | | | | | |

**Key**

|  | Statistically significant, intervention/exposure beneficial |
| --- | --- |
|  | Statistically significant, intervention/exposure non-beneficial |
|  | Not statistically significant |
|  | Statically significant, only where baseline levels indicate poor health |

**Supplementary Table 9. ROBINS-I V2 Risk of Bias Tool for C-RCT**

| **Study** | **S** | **Domain 1** | **Domain 2** | **Domain 3** | **Domain 4** | **Domain 5** | **Domain 6** | **Domain 7** | **Overall risk of bias** |  |  |  |  |  |  |  |  |
| --- | --- | --- | --- | --- | --- | --- | --- | --- | --- | --- | --- | --- | --- | --- | --- | --- | --- |
| Grant et al, 2017(a) [2] |  |  |  |  |  |  |  |  |  |  |  |  |  |  |  |  |  |
| Bender-Mendl et al, 2022 [3] |  |  |  |  |  |  |  |  |  |  |  |  |  |  |  |  |  |
| Hampton et al, 1996 [7] |  |  |  |  |  |  |  |  |  |  |  |  |  |  |  |  |  |
| Leung et al, 2021 [8] |  |  |  |  |  |  |  |  |  |  |  |  |  |  |  |  |  |
| HolmbÃ¤ck et al, 2003 [9] |  |  |  |  |  |  |  |  |  |  |  |  |  |  |  |  |  |
| Bonham et al, 2020 [10] |  |  |  |  |  |  |  |  |  |  |  |  |  |  |  |  |  |
| Al-Naimi et al, 2004 [11] |  |  |  |  |  |  |  |  |  |  |  |  |  |  |  |  |  |
| Grant et al, 2017(b) [12] |  |  |  |  |  |  |  |  |  |  |  |  |  |  |  |  |  |
| Silva et al, 2020 [14] |  |  |  |  |  |  |  |  |  |  |  |  |  |  |  |  |  |
| Teixeira et al, 2022 [15] |  |  |  |  |  |  |  |  |  |  |  |  |  |  |  |  |  |
| Gupta et al, 2021 [16] |  |  |  |  |  |  |  |  |  |  |  |  |  |  |  |  |  |
| Leedo et al, 2017 [18] |  |  |  |  |  |  |  |  |  |  |  |  |  |  |  |  |  |
| Oriyami et al, 2021 [19] |  |  |  |  |  |  |  |  |  |  |  |  |  |  |  |  |  |
| Centofanti et al, 2018 [24] |  |  |  |  |  |  |  |  |  |  |  |  |  |  |  |  |  |
| Gupta et al, 2017 [25] |  |  |  |  |  |  |  |  |  |  |  |  |  |  |  |  |  |
| Love et al, 2005 [26] |  |  |  |  |  |  |  |  |  |  |  |  |  |  |  |  |  |
| Paz & Berry, 1997 [27] |  |  |  |  |  |  |  |  |  |  |  |  |  |  |  |  |  |
| Morris et al, 2016 [30] |  |  |  |  |  |  |  |  |  |  |  |  |  |  |  |  |  |
| Jenson et al, 2020 [35] |  |  |  |  |  |  |  |  |  |  |  |  |  |  |  |  |  |
| Knutsson et al, 2002 [41] |  |  |  |  |  |  |  |  |  |  |  |  |  |  |  |  |  |
| Lowden et al, 2004 [43] |  |  |  |  |  |  |  |  |  |  |  |  |  |  |  |  |  |
| **Footnotes:** Green, diagonal left down: Low risk of bias. Yellow, grid: Some concerns. Red, horizontal lines: High risk of bias. Dark red, diagonal right up: Critical risk. Grey, solid: N/A.  S: Screening / decide whether to proceed with a risk-of-bias assessment; Domain 1: Bias due to confounding; Domain 2: Bias in classification of interventions; Domain 3: Bias in selection of participants into the study; Domain 4: Bias due to deviations from intended interventions; Domain 5: Bias due to missing data; Domain 6: Bias in measurement of the outcome; Domain 7: Bias in selection of the reported result. | | | | | | | | | |  |  |  |  |  |  |  |  |

**Supplementary Table 10. Newcastle-OTTOWA Risk of Bias Tool for Observational Studies**

| **Study** | **Selection Domain**  **(max 4 stars)** | **Comparability Domain**  **(max 1 star)** | **Outcome Domain**  **(max 3 stars)** | **Overall risk of bias** |
| --- | --- | --- | --- | --- |
| Rodrigues et al, 2022 [13] | 1 | 0 | 1 |  |
| Park et al, 2023 [20] | 0 | 1 | 0 |  |
| Molzof et al, 2022 [21] | 1 | 0 | 2 |  |
| Khorasaniha et al, 2022 [22] | 1 | N/A | 0 |  |
| O'brien et al. 2020 [23] | 1 | N/A | 1 |  |
| Kulak and Yesil 2022 [29] | 1 | N/A | 1 |  |
| Lowden et al, 2001 [31] | 1 | 1 | 2 |  |
| Rijk et al, 2021 [33] | 3 | 0 | 3 |  |
| Jung et al, 2020 [34] | 3 | 1 | 2 |  |
| Wirth et al, 2015 [36] | 3 | 1 | 2 |  |
| Wirth et al, 2022 [37] | 3 | 1 | 3 |  |
| Khorasaniha et al, 2020 [39] | 1 | 1 | 2 |  |
| Molzof et al, 2017 [40] | 3 | 1 | 1 |  |
| Kräuchi et al, 1990 [42] | 2 | 1 | 2 |  |
| **Footnotes:** Green, diagonal left down: Good quality. Yellow, grid: Fair quality. Red, horizontal line: Poor quality. Grey, solid: N/A  Thresholds for converting the Newcastle-Ottawa scales to AHRQ standards (good, fair, and poor): Good quality: 3 or 4 stars in selection domain AND 1 or 2 stars in comparability domain AND 2 or 3 stars in outcome/exposure domain; Fair quality: 2 stars in selection domain AND 1 or 2 stars in comparability domain AND 2 or 3 stars in outcome/exposure domain; Poor quality: 0 or 1 star in selection domain OR 0 stars in comparability domain OR 0 or 1 stars in outcome/exposure domain | | | | |

**Supplementary Table 11. COCHRANE Risk of Bias Tool for RCT**

| **Study** | **Domain 1** | **Domain 2** | **Domain 3** | **Domain 4** | **Domain 5** | **Overall Risk of Bias** |
| --- | --- | --- | --- | --- | --- | --- |
| Manoogian et al, 2022 [1] |  |  |  |  |  |  |
| Gupta et al, 2019(a) [3] |  |  |  |  |  |  |
| Gupta et al, 2019(b) [4] |  |  |  |  |  |  |
| Nehme et al, 2014 [5] |  |  |  |  |  |  |
| Cunha et al, 2020 [6] |  |  |  |  |  |  |
| Makowski et al, 2022 [17] |  |  |  |  |  |  |
| Chellappa et al, 2021 [28] |  |  |  |  |  |  |
| Smith-Ryan, 2019 [38] |  |  |  |  |  |  |
| **Footnotes:** Green, diagonal left down: Low risk of bias. Yellow, grid: Some concerns. Red, horizontal line: High risk of bias. Domain 1: Risk of bias arising from the randomisation process; Domain 2: Risk of bias due to deviations from the intended interventions; Domain 3: Risk of bias due to missing outcome data; Domain 4: Bias in measurement of the outcome; Domain 5: Bias in selection of the reported result. | | | | | | |
